# Isolation and transcriptional characterization of mouse perivascular astrocytes

**DOI:** 10.1371/journal.pone.0240035

**Published:** 2020-10-08

**Authors:** Nejla Yosef, Yuanxin Xi, Joseph H. McCarty

**Affiliations:** 1 Department of Neurosurgery, University of Texas M.D. Anderson Cancer Center, Houston, TX, United States of America; 2 Department of Bioinformatics and Computational Biology, University of Texas M. D. Anderson Cancer Center, Houston, TX, United States of America; Medical College of Wisconsin, UNITED STATES

## Abstract

In the post-natal mammalian brain perivascular astrocytes (PAs) ensheath blood vessels to regulate their unique permeability properties known as the blood-brain barrier (BBB). Very little is known about PA-expressed genes and signaling pathways that mediate contact and communication with endothelial cells (ECs) to regulate BBB physiology. This is due, in part, to lack of suitable models to distinguish PAs from other astrocyte sub-populations in the brain. To decipher the unique biology of PAs, we used in vivo gene knock-in technology to fluorescently label these cells in the adult mouse brain followed by fractionation and quantitative single cell RNA sequencing. In addition, PAs and non-PAs were also distinguished with transgenic fluorescent reporters followed by gene expression comparisons using bulk RNA sequencing. These efforts have identified several genes and pathways in PAs with potential roles in contact and communication with brain ECs. These genes encode various extracellular matrix (ECM) proteins and adhesion receptors, secreted growth factors, and intracellular signaling enzymes. Collectively, our experimental data reveal a set of genes that are expressed in PAs with putative roles in BBB physiology.

## Introduction

The blood-brain barrier (BBB) is formed and maintained through complex interactions between cells of the neurovascular unit (NVU), which is comprised of endothelial cells (ECs), pericytes, astrocytes, microglia and neurons [1, 2]. Various glial-derived pathways have been identified that promote the formation of the BBB during embryonic and neonatal development [3]. For example, vascular endothelial growth factor-A (VEGF) expressed by glial cells signals via the VEGFR2 receptor tyrosine kinase in ECs to control blood vessel sprouting and permeability [4]. In addition, Wnt growth factors secreted by glial cells promote angiogenesis and barriergenesis [5] via β-catenin activation in ECs [6]. Wnt signaling also involves GPR124, an atypical G-protein coupled receptor expressed in ECs that is essential for vascularization [7, 8] via interactions with the Wnt co-receptor Reck [9]. Integrin-mediated activation of TGFβ signaling is also critically involved in control of angiogenesis and EC barrier development [10–13].

Astrocytes are an abundant cell type in the post-natal central nervous system (CNS) and have many important physiological functions at the NVU [14, 15]. In vitro models support roles for mixed cultures of astrocytes in mediating the induction and maintenance of the BBB by controlling EC barrier characteristics including tight junction protein expression and transendothelial endocytosis [16]. However, astrocytes are highly heterogeneous and display variability related to differences in their lineage and regional location in the brain [17, 18]. Thus, most *in vitro* co-culture models likely lack accurate features involved in astrocyte contact and communication with ECs in vivo. PA end feet juxtapose nearly 80% of the abluminal surface of capillaries, revealing a crucial role for these cells in BBB physiology. Indeed, PA adhesion and signaling pathways are known to play important roles in regulation of the BBB. For example, the dystrophin-glycoprotein complex is enriched in PAs [19] and has roles in regulating BBB development and integrity [20]. Genetic deletion of the DGC component aquaporin 4 (*Aqp4*) in astrocytes leads to abnormal interactions with endothelial cells and impaired BBB integrity [21]. In addition, hedgehog produced by PAs is essential for normal control of BBB physiology via signaling through smoothened receptors in the vascular endothelium [22]. TGFβ signaling promotes expression of Mfsd2a in post-natal brain ECs [23]. Mfsd2a transports the omega-3 fatty acid docosahexaenoic acid (DHA) across the BBB and promotes BBB homeostasis, with genetic deletion of *Mfsd2a* in mice leading to BBB hyperpermeability and DHA deficiency [24, 25]. Loss-of-function mutations in human MFSD2A are linked to cognitive coordination impairment due to deficiencies in DHA metabolism [26]. In addition to these various EC-intrinsic signaling events, PAs also control blood vessel functions through communication with pericytes [27]. For example, pericyte interactions with ECs via the platelet derived growth factor B pathway is important for BBB integrity [28]. PAs also regulate brain homeostasis through modulation of the glymphatic system [29].

Additional mechanistic insights about how PAs regulate the BBB have been hindered by the lack of suitable *in vivo* models that distinguish PAs from other astroglial cell populations. Knock-in and transgenic strategies in mice commonly use promoters from glial fibrillary acidic protein (*Gfap*) [30], aldehyde dehydrogenase 1 family member L1 (*Aldh1l1*) [31], and glutamate aspartate transporter (*Slc1a3*) [32] genes to visualize and manipulate astrocytes. However, many of these models do not distinguish the diverse sub-populations of astrocytes found throughout the brain. Therefore, the selective identification, isolation and characterization of PAs is necessary for determining the full potential of PA-derived signaling pathways. In order to identify PA-expressed genes and pathways, we have focused efforts on the gene encoding megalencephalic leukoencephalopathy with subcortical cysts 1 (*Mlc1*), a transmembrane protein that is enriched in PA end feet [33]. Using T2A ribosome skipping strategies, we generated an mouse knock-in model in which the *Mlc1* gene drives the expression of enhanced green fluorescent protein (Mlc1-EGFP) [34] without perturbing expression of endogenous Mlc1 protein. Here, we have fractionated PAs from the brains of Mlc1-EGFP mice and analyzed their gene expression by quantitative single cell RNA sequencing. We also compared differential gene expression levels in PAs versus non-PAs and validated several of these genes as highly PA-enriched. Collectively, these data provide an important resource to the glial cell biology community and may reveal novel roles for PAs in the regulation of blood vessel functions, and particularly the BBB, in the adult brain.

## Materials and methods

### Experimental mice

This study was reviewed and approved by the MD Anderson Cancer Center Institutional Animal Care and Use Committee (IACUC) in compliance with the National Research Council Guide for the Care and Use of Laboratory Animals. The approved protocol number is ACUF-00001108-RN02. Mlc1-EGFP knock-in mice were generated as previously described [34]. Mlc1-EGFP/Mlc1-EGFP homozygous knock-in animals were crossed to homozygous GLAST-DsRed^tg/tg^ transgenic mice [35] to generate Mlc1-EGFP/^+^;GLAST-DsRed^tg^/^+^ double heterozygous F1 progeny (Mlc1-EGFP/^+^;GLAST-DsRed^tg^/^+^). All knock-in mice were on a mixed C57Bl6/129S1/FVB background. All animals were genotyped using PCR-based methodologies as reported previously [34, 35]. Mice were analyzed between postnatal day 30 (P30) and P90. We have reported previously that EGFP is stably expressed in perivascular astrocytes in the adult brain beyond P30 [34]. Since there were no reported sex-dependent differences in EGFP or DsRed expression in the two models, males and females were often combined for genomic or immunohistochemical analysis.

### scRNAseq experiments

Cerebral cortices were dissected from three adult Mlc1-EGFP/^+^ mice of similar age and placed in ice-cold PBS. In a tissue culture hood, cortices were minced using a sterile razor blade in a 60 mm petri dish containing sterile ice-cold 1X HBSS. After rapid transfer into a polypropylene tube, the suspension was centrifuged at 4°C and 300 g for 5 minutes. Cortical pellets were digested with an enzyme cocktail for 15 minutes using the Brain Tumor Dissociation Kit (#130-096-942) following the manufacturer guidelines (Miltenyi Biotec, Inc.). The suspension was gently mixed with a 5 ml pipette. The enzyme activity was terminated by addition of an equal volume of cold 1X HBSS. The suspension was gently mixed by pipetting several times with a 5 ml pipette and filtered through a 70 μm cell strainer into clean tubes to generate single cell suspensions. A live cell count was determined using the 0.4% trypan blue exclusion test, and enumeration was performed with a hemocytometer. Samples were centrifuged and resuspended with cold PBS containing 5% FBS at a concentration of 5x10^6^ cells/ml. Samples were then transferred into polypropylene fluorescence-activated cell sorting (FACS) tubes for cell sorting by EGFP signal using a BD FACS Aria II cell sorter. GFP positive cells were sorted using a 100 μm nozzle and captured in a chilled tube containing PBS with 5% FBS. Fresh cell suspensions were immediately subjected to the scRNAseq protocol.

FAC-sorted PAs (EGFP^+^ cells) from pooled cortices of three adult Mlc1-EGFP/+ mice were counted before being loaded onto the 10X Genomic Chromium instrument. The scRNAseq libraries were generated using the 10X Chromium Single Cell 3’ v2 reagent kit and were sequenced on a Nextseq500 instrument. Sequencing data were analyzed using 10X Genomic Cell Ranger software. Expression matrices were processed by the SEURAT package v2.3.1 [36]. First, the raw UMI counts were normalized and scaled using the “NormalizeData” and “ScaleData” function in SEURAT. Principal component analysis was performed using highly variable identified by the “FindVariableGenes” function with default parameters, and significant principal components were selected to represent at least 80% of the total variance. Using these significant principle components, the UMAP plot was generated for two-dimensional visualization of multi-dimensional dataset. Clustering analyses were performed using the “FindCluster” function in SEURAT, with resolution set to 0.7 and reduction type set to “umap”. For each cluster, the marker genes were identified by comparing gene expression in the cluster vs other clusters, using Wilcoxon Rank-Sum test with adjusted p-value cutoff 0.05. To avoid sample size bias caused by different number of cells in each cluster, we sub sampled 50 cells from each cluster for the comparisons.

### Bulk RNAseq experiments

Cerebral cortices were dissected from the brains of adult Mlc1-EGFP;GLAST-DsRed mice (n = 4) of similar age (P30) in ice-cold PBS. Cerebral cortices from each mouse were dissociated separately into a single cell suspension as described above. A live cell count was determined using the 0.4% trypan blue exclusion test, and enumeration was performed with a hemocytometer. Samples were centrifuged and resuspended with cold PBS at a concentration of 5x10^6^ cells/ml. Propidium iodide was added to suspensions prior to sorting to distinguish non-viable cells and cellular debris. Cell doublets were excluded from the sorted fractions by standard forward scatter and side scatter analyses. Gates for sorting PAs (EGFP^+^;DsRed^+^) and non-PAs (DsRed^+^ cells) from Mlc1-EGFP;GLAST-DsRed double transgenic mice were determined using cell suspensions from Mlc1-EGFP or GLAST-DsRed cortical tissue. Samples were then transferred into FACS tubes for cell sorting by DsRed and GFP signals using a BD FACSAria II cell sorter. PAs (EGFP^+^;DsRed^+^) and non-PAs (DsRed^+^ cells) were sorted using a 100 μm nozzle and captured in a chilled tube containing PBS. Freshly sorted cell suspensions were immediately subjected to quantitative RNA sequencing to compare gene expression profiles. As gating controls for sorting, we used cells from dissociated cerebral cortices taken from adult Mlc1-EGFP mice and GLAST-DsRed mice.

FAC-sorted PAs *(EGFP*^+^/*DsRed*^+^ cells) or non-PAs (*DsRed*^*+*^ cells) from mouse tissue (n = 4 mice) were pelleted and the total RNA was extracted following the Qiagen RNeasy micro kit guidelines. After RNA quality was validated, eight samples of a RIN of ≥ 7 were loaded onto HiSeq400 instrument. Each astrocyte population was sequenced in quadruplicates. An average of ~43 million paired-end reads were generated for each of the eight samples. Sequenced reads fastq files were mapped to mouse reference genome GRCm3 (mm10).using Tophat v2.0.13 [37]. Raw reads count were calculated using HTseq 0.12.4 [38]. Differentially expressed genes between *EGFP*^+^*/DsRed*^+^ and *DsRed*^*+*^ cells were obtained with EdgeR package [39]. Differentially expressed genes are those having logFC > 2 and FDR< 0.01. Expression heat maps were generated using the differentially expressed genes. Volcano plots were generated using adjusted p-value (FDR) and log2 fold changes. Gene set enrichment analysis was performed using the GSEA software [40]. The lists of enriched KEGG pathways were also compiled from GSEA results. For cross-referencing the PA 10 gene signature as well as 5 canonical astrocyte markers, we downloaded the normalized expression data matrix data for GSE72826 [18], and generated a heatmap showing relative expression levels of the selected genes. Hierarchical clustering analysis was performed to explore the expression patterns of these signature genes.

### Quantitative RT-PCR

Freshly sorted PAs (EGFP^+^ or EGFP^+^/DsRed^+^ cells) and non-PAs (DsRed^+^ cells) were washed with cold PBS and the total RNA was extracted following the Qiagen RNeasy micro kit guidelines. RNA was reverse transcribed using Invitrogen SuperScript® III cDNA synthesis kit, and 100ng cDNA was used per reaction. qRT-PCR was carried out using Taqman primers, Taqman Universal Master Mix II on a StepOnePlus Real-Time PCR system (Applied Biosystems). The following Taqman qRT-PCR primers were used: Megalencephalic Leukoencephalopathy With Subcortical Cysts 1 (*Mlc1*, Mm00453827_m1), Glial high affinity glutamate transporter (*Slc1a3*, Mm00600697_m1), Aldehyde dehydrogenase 1 family member L1 (*Aldh1l1*, Mm03048957_m1), Glial fibrillary acidic protein (GFAP, Mm01253033_m1), Integrin subunit alpha-7 (*Itga7*, Mm00434400_m1), laminin subunit alpha 5 (*Lama5*, Mm01222029_m1), Semaphorin 4A (*Sema4a*, Mm00443140_m1), Semaphorin 4B (*Sema4b*, Mm00803797_m1), Four-jointed box protein 1 (*Fjx1*, Mm00487385_s1), Growth differentiation factor 10 (*Gdf10*, Mm01220860_m1), Gap junction beta-2 protein (*Gjb2*, Mm00433643_s1), Follistatin-related protein 1 (*Fstl1*, Mm00433371_m1), and Ectonucleoside triphosphate diphosphohydrolase 2 (*Entpd2*, Mm00515450_m1). *18S* (4333760F) or *Gapdh* (Mm99999915_g1) were used for normalization and relative gene expression ratios were calculated using quantitative ΔCT methods.

Statistical analysis of qRT-PCR was performed using GraphPad prism 6 software. Differences between groups were analyzed using two-way analysis of variance (ANOVA) with Bonferroni post-hoc test and with the following denotations for statistical significance: *p<0.05, **p<0.01, ***p<0.001, ****p<0.0001.

### Immunofluorescence and confocal microscopy

Adult mice were anesthetized and fixed by cardiac perfusion with 4% paraformaldehyde (PFA) in PBS. Brains were removed and immersed in 4% PFA for 24 hours at 4°C. After fixation, brains were washed with cold 1XPBS 3 times for 10 minutes at 4°C. Then fixed brains were sagittally sectioned and embedded in 4% agarose. Then brains were sectioned at 100 μm on a vibratome and stored in 1X PBS at 4°C. For immunofluorescence staining, sections were permeabilized and blocked with 1XTBS supplemented with 0.1% Triton-X and 10% donkey serum for 1 hour at room temperature (RT), followed by incubation with primary unconjugated antibodies diluted in the blocking solution and incubated overnight at 4°C. Immunofluorescence analyses were performed with the following primary antibodies: GFP, RFP, GFAP, and CD31 (S1 Table). These sections were then washed 3 times with TBS for 5 minutes and incubated with secondary antibodies (1:400 dilution), and 4'-6-Diamidino-2-phenylindole (DAPI) (1:500 dilution) in the blocking solution for 1 hour at RT (S1 Table). After washing with 1XTBS as previously stated sections were briefly washed with 1XPBS. Then sections were mounted on pre-treated microscope slides and sealed using vectashield (Vector Laboratories, Inc., Burlingame, Ca) mounting media and kept at 4°C until imaging. Confocal Images were acquired using an Olympus FLUOVIEW FV1000 20X and 40X objectives. All comparative images were taken with the same laser power and gain settings in order to make qualitative comparisons between staining levels in different samples. Multiple fields of view were imaged from biological replicates. The ratio of EGFP^+^ cells, *DsRed*^+^ cells or EGFP^+^/DsRed^+^ cells was calculated with respect to total number of cells in a field of view. The total number of cells in the field of view was calculated by enumerating the number of DAPI stained nuclei using Adobe Photoshop CC (Adobe Systems, Inc.).

## Results

### Visualization of PAs in the cerebral cortex of Mlc1-EGFP mice

**Fig 1:**
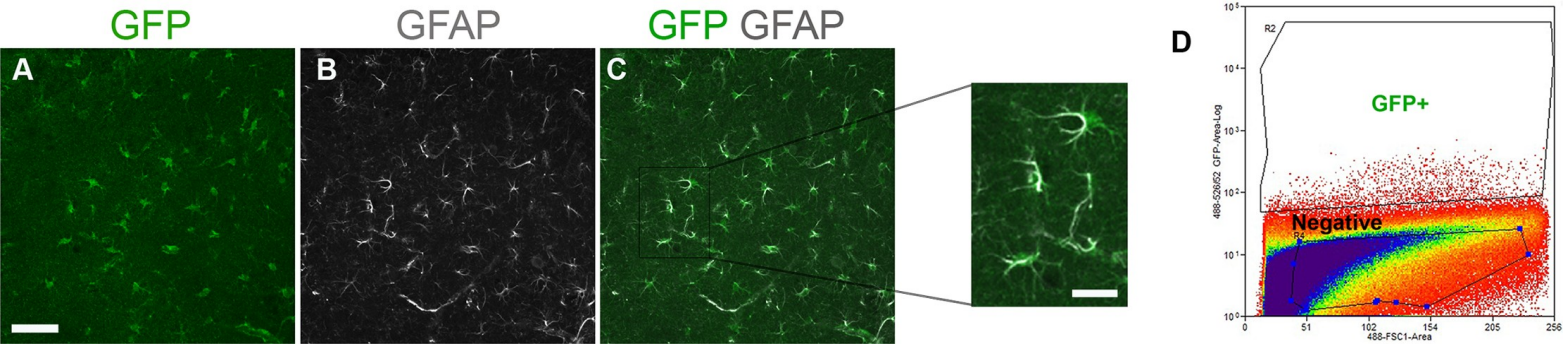
Fractionation of PAs from the cerebral cortices of adult Mlc1-EGFP mice. **(A–C)**; Sagittal brain sections through the cerebral cortices of P30 mice were double immunolabeled with anti-GFP (A) and anti-GFAP (B) antibodies. Note the co-expression of GFP and GFAP in astrocytes (C). Scale bars, 50 μm (A) and 10 μm (inset, C). **(D);** Flow cytometric analyses of dissociated cerebral cortical cells from Mlc1-EGFP mice reveals that a low percentage of cells express EGFP (EGFP^+^). Note that the vast majority of cells do not express EGFP (Negative).

To characterize the molecular properties of brain PAs, we utilized the Mlc1-EGFP knock-in mouse model. In this model the *Mlc1* gene drives EGFP reporter expression via a T2A insertion without impacting endogenous Mlc1 protein expression [34]. Prior to fractionating -EGFP^+^ cells from adult mice, we first confirmed the spatial distribution of EGFP^+^ cells in the brain. The expression of EGFP in unfixed tissue was only weakly detected; therefore, we used an anti-GFP antibody in combination with an antibody directed against glial fibrillary acidic protein (GFAP) to reveal co-localization of EGFP and GFAP in astrocytes (Fig 1A–1C). These results are consistent with our prior report showing that EGFP^+^ cells in Mlc1-EGFP mice express GFAP and are closely associated with brain blood vessels [34]. To selectively isolate and characterize PAs from the brain, cerebral cortices from adult Mlc1-EGFP mice were enzymatically digested and EGFP^+^ cells were isolated using fluorescent activated cell-sorting (FACS) approaches. Despite the very low level of fluorescent signal detectable in fixed brain tissue slices, we detected EGFP by live cell sorting and successfully fractionated PAs from the cerebral cortices (Fig 1D). Thus, fluorescence-based sorting allows for the efficient identification and isolation of live PA cells from the adult Mlc1-EGFP mouse brain.

### Single cell RNA sequencing analysis to identify genes with enriched expression in PAs

**Fig 2:**
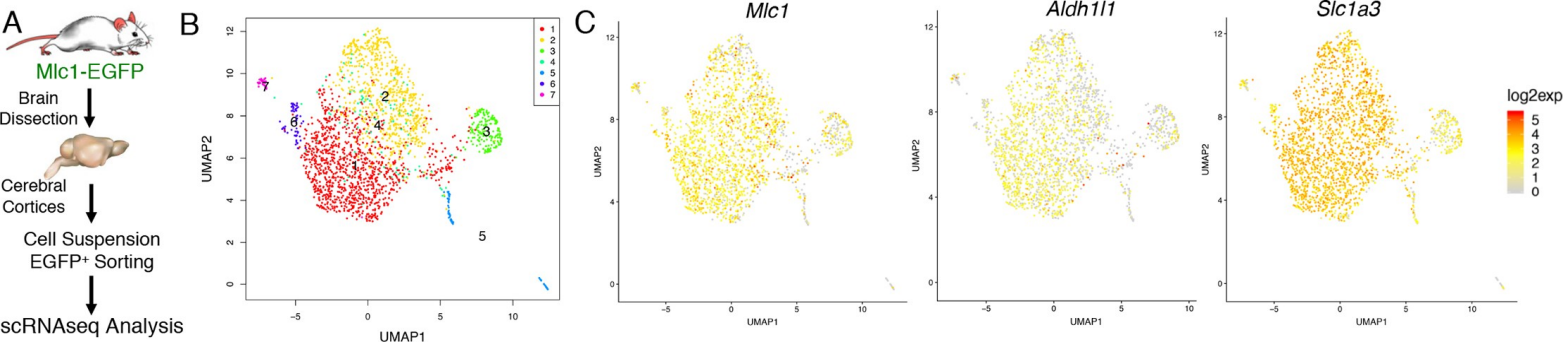
Analysis of gene expression in PAs isolated from the cerebral cortices of adult Mlc1-EGFP mice. **(A);** Schematic summarizing the experimental strategies for isolating cells from cerebral cortices of adult (P30) Mlc1-EGFP knock-in mice. Brain tissue was dissociated and live PAs were fractionated based on EGFP expression using FACS-based approaches. Single cells were subsequently analyzed using quantitative RNA sequencing. **(B);** Unsupervised clustering of single cell RNA sequencing data using EGFP-expressing PA cells fractionated from mouse cerebral cortices. Cells are clustered based on shared similarities in mRNA expression, with each of the two main clusters representing a subpopulation of PAs showing differential, but somewhat overlapping gene expression. **(C);** Feature plots showing the expression of select genes with established roles in mouse brain astrocytes including *Mlc1 Aldh1l1*, and *Slc1a3*. Each dot indicates a distinct mRNA sequencing result from a single cell. Red indicates higher gene expression and grey indicates lower gene expression.

We next fractionated PAs from the cortices of adult Mlc1-EGFP mice (Fig 2A) followed by quantitative single cell RNA sequencing (scRNAseq). After computational comparisons, we performed graph-based clustering on all significant components. As a result, we detected seven distinct cell clusters, with the bulk of cells segregating into two main clusters with overlap due to similarities in mRNA expression (S1 Fig), although unbiased expression analyses of cell clusters 1 and 2 revealed some differences in gene expression (S2 Fig). The scRNAseq data were also visualized through a non-linear dimensional reduction algorithm (Fig 2B). Expression of well-established astrocyte transcripts such as *Mlc1*, *Slc1a3* and *Aldh1l1* (Fig 2C) supported the astrocytic identity of PA fractions. In contrast, established markers for brain vascular ECs such as *Cldn5* were expressed at low or undetectable levels in clusters 1 and 2 (S3 Fig). Importantly, the scRNAseq data showed that PAs, and especially clusters 1 and 2, were enriched in mRNAs with published roles in vascular biology (S3 Fig). These enriched genes encode integrin beta8 (*Itgb8*) which activates TGFβ signaling in brain vascular endothelial cells [41] and vascular endothelial growth factor-A (*Vegfa*), which is expressed by astrocytes and regulates blood vessel permeability in the brain [42]. The top 20 most enriched genes in each of the 7 isolated cell clusters are summarized in the Supplemental Materials section (S2–S8 Tables).

### Analyzing gene expression profiles in PAs versus non-PAs by RNAseq

**Fig 3:**
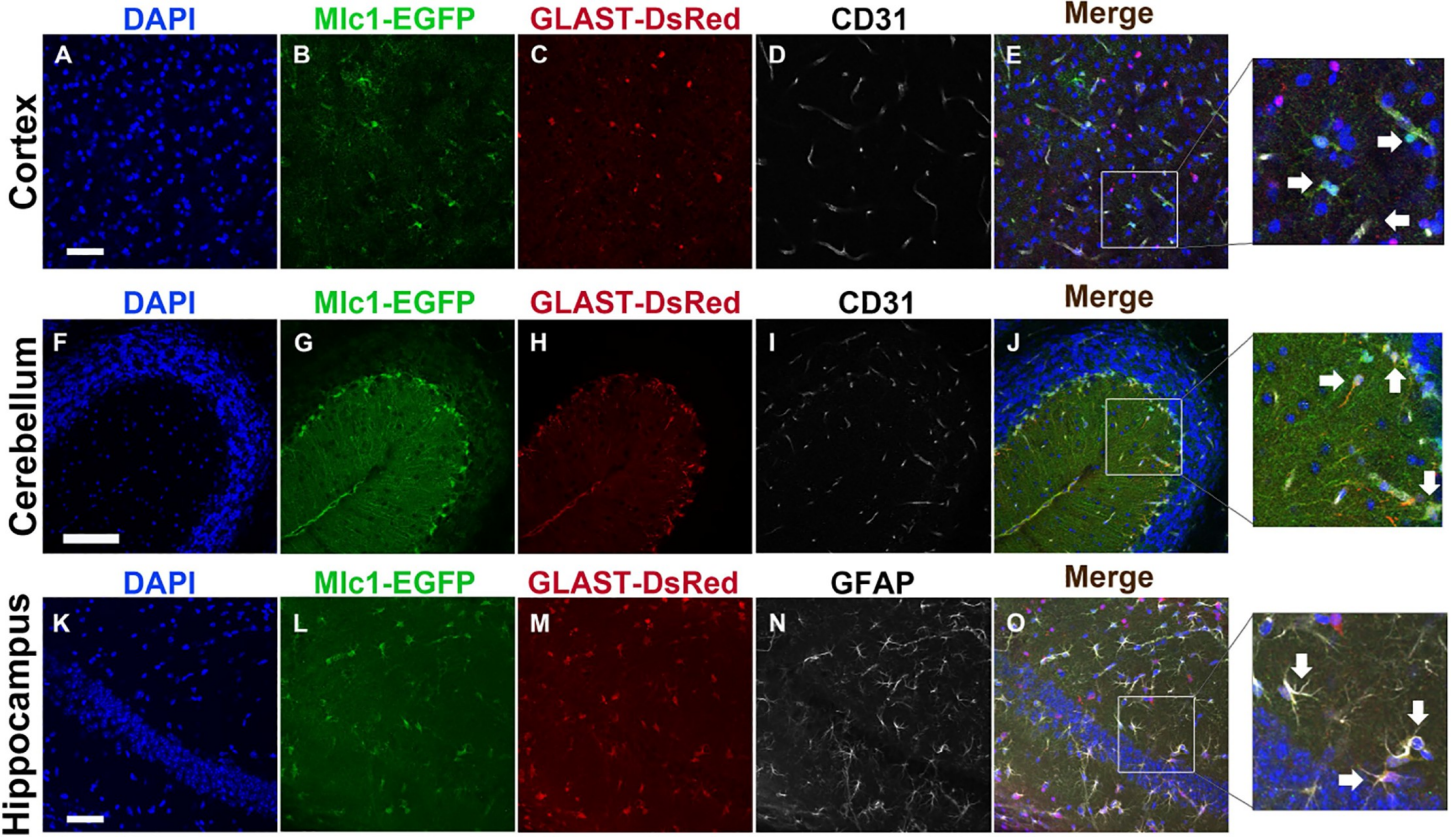
Analyzing PA and non-PA cell distribution in adult Mlc1-EGFP;GLAST-DsRed mice based on EGFP and DsRed reporter expression. **(A-E);** Coronal brain sections through the cerebral cortices of an adult Mlc1-EGFP; GLAST-DsRed mouse brain were labeled with DAPI (A), anti-GFP to detect PAs (B), anti-RFP to detect DsRed-expressing PAs and non-PAs (C), and anti-CD31 to detect vascular ECs that comprise blood vessels (D). Note that cells adjacent to cortical blood vessels (PAs) co-express GFP and DsRed (arrows in E), whereas DsRed^+^ cells (non-PAs) that are more distal to CD31^+^ ECs lack GFP expression. Scale bars, 100 μm (A) and 20 μm (inset, E). **(F-J);** Coronal sections through the cerebellum of an adult Mlc1-EGFP;GLAST-DsRed mouse brain stained with DAPI (F), anti-GFP (G), anti-RFP (H), and anti-CD31 (I). Note that most if not all cerebellar Bergmann glial cells, which are a radial glial-like astrocyte sub-population, co-express GFP and DsRed (arrows in J). Scale bars, 100 μm (E) and 20 μm (inset, J). **(K-O)**; Coronal sections through the hippocampus of an adult Mlc1-EGFP;GLAST-DsRed mouse brain labeled with DAPI (K), anti-GFP (L), anti-RFP (M), and anti-GFAP (N). Note that many hippocampal astrocytes co-express GFP, DsRed, and GFAP (arrows in O). Scale bars, 100 μm (K) and 20 μm (inset, O).

**Fig 4:**
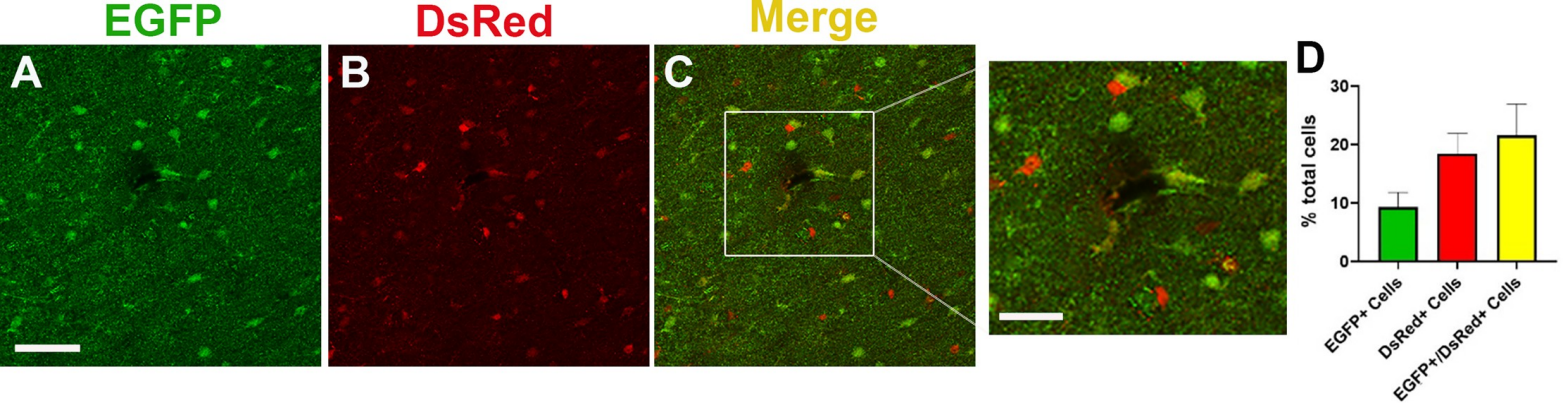
Quantitation of PAs and non-PAs within the cerebral cortices of Mlc1-EGFP;GLAST-DsRed adult mice. **(A-C);** Coronal brain sections through the cerebral cortices of adult Mlc1-EGFP;GLAST-DsRed mice were immunolabeled with anti-GFP antibodies (A) to visualize Mlc1-expressing cells (PAs) and anti-RFP antibodies (B) to visualize cells expressing DsRed via the *Slc1a3* promoter (PAs and non-PAs). In the merged confocal image (C), note that some cells express both EGFP^+^ and DsRed^+^, whereas other cells are single positive for each fluorescent marker. Scale bar, 50 μm (A) and 10 μm (inset, C). **(D);** Quantification of the EGFP^+^ and RFP^+^ single positive cells as well as EGFP^+^/RFP^+^ double positive cells in the cortices of Mlc1-EGFP;GLAST-DsRed mice. Numbers of fluorescence positive cells are plotted as a percentage of total numbers of cells based on DAPI labeling, although DAPI images have not been included in this figure. Note that more than 20% of cells within the adult cerebral cortex are EGFP^+^ or EGFP^+^/RFP^+^, whereas non-PAs constitute approximately 20% or more of total cells. The red bars indicate DsRed^+^/EGFP^-^ cell fractions.

To compare the gene expression profiles of PAs versus other astrocyte populations (non-PAs) in the brain that do not express Mlc1, we interbred Mlc1-EGFP homozygous knock-in mice with GLAST-DsRed transgenic mice [35] to generate Mlc1-EGFP;GLAST-DsRed F1 progeny. As detailed above, PAs express EGFP in the Mlc1-EGFP brain, whereas GLAST-DsRed transgenic mice express DsRed in many subsets of astrocytes, including PAs, throughout the brain via the astrocyte specific glutamate transporter (*Slc1a3*) promoter [35]. To confirm the astrocyte-specific expression patterns of EGFP and DsRed, we first labeled brain sections with antibodies recognizing GFP to identify PAs and RFP to identify PAs and non-PAs. In addition, anti-CD31 or anti-GFAP antibodies were included to identify vascular ECs or astrocytes, respectively. Our data showed that EGFP^+^ and DsRed^+^ single positive cells as well as double positive (EGFP^+^/DsRed^+^) cells were found throughout the cortex, cerebellum and hippocampus (Fig 3A–3C, 3F–3H and 3K–3M). Importantly, cells adjacent to blood vessels showed EGFP and DsRed co-expression, that were distinct from CD31 expressing vascular ECs (Fig 3D, 3E, 3I and 3J). Additionally, most cells showing co-expression of EGFP and DsRed were also positive for GFAP (Fig 3N and 3O). To more precisely determine the distribution of PAs and non-PAs in the cerebral cortex, we analyzed Mlc1-EGFP;GLAST-DsRed brain sections by immunolabeling with anti-GFP and anti-DsRed antibodies (Fig 4A–4C). Quantitation of the GFP, DsRed, and GFP/DsRed positive cells revealed that <10% were EGFP^+^, ~18% were DsRed^+^, and ~20% were EGFP^+^/DsRed^+^ (Fig 4D).

**Fig 5:**
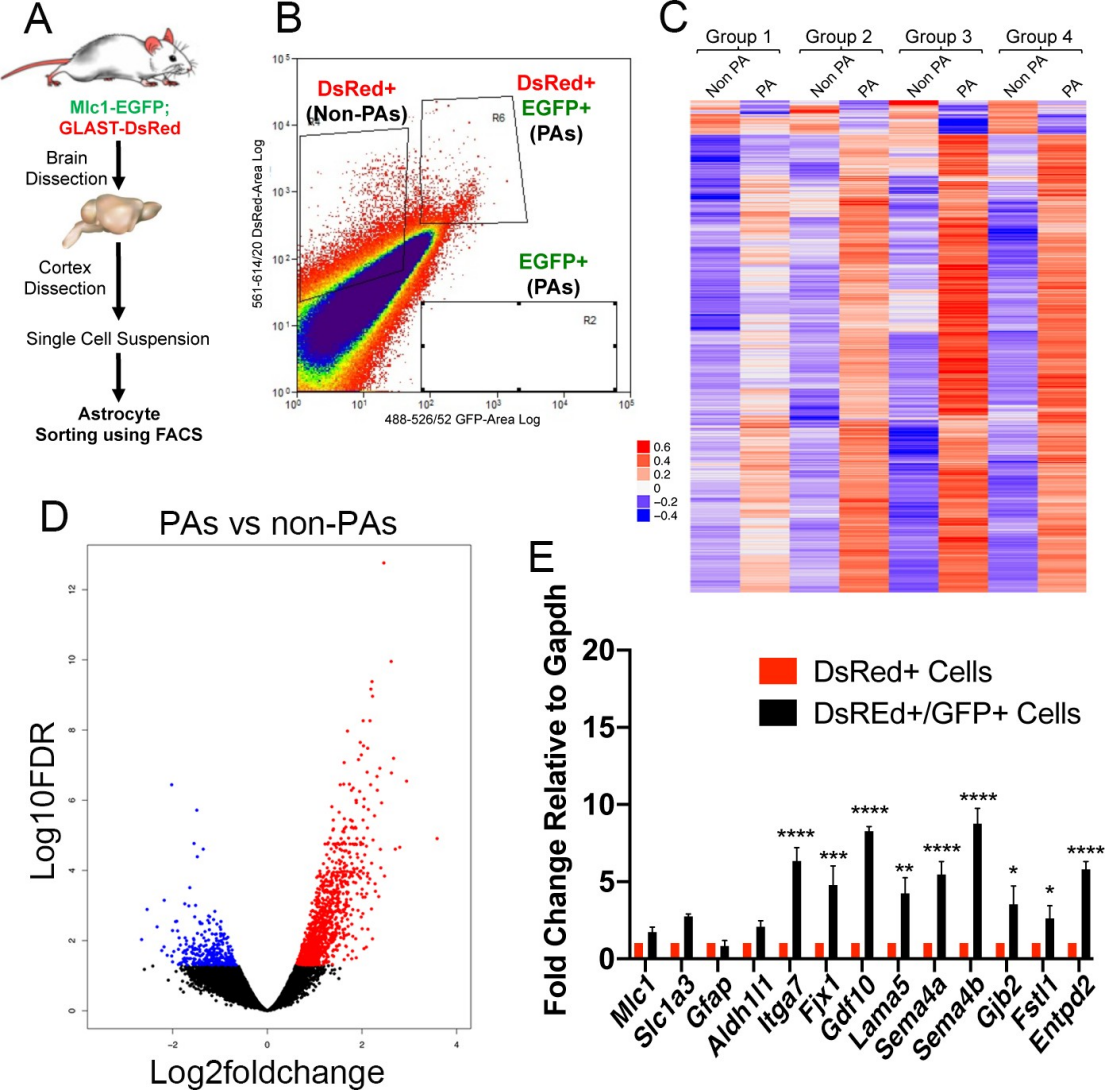
Quantitative RNA sequencing to identify differentially expressed genes in PAs versus non-PAs isolated from Mlc1-EGFP;GLAST-DsRed adult mice. **(A);** Strategy for dissection and analysis of cells from cerebral cortices of Mlc1-EGFP;GLAST-DsRed adult mice. Non-PAs (DsRed^+^ cells) and PAs (EGFP^+^/DsRed^+^ cells) were fractionated using FAC-based sorting approaches followed by comparative RNA sequencing. **(B);** Flow cytometry plot showing the profile of sorted DsRed^+^ single positive cells (non-PAs) and EGFP^+^/DsRed^+^ double positive cells (PAs) from the cerebral cortices. Note that the majority of cells are DsRed^+^ and nearly all EGFP^+^ cells are double positive for DsRed. The few cells in the lower box are EGFP^+^ single positive PAs that lack DsRed expression. **(C, D);** Quantitative RNA sequencing comparisons reveal differentially expressed genes in PAs versus non-PAs as revealed by a color-coded heat map (C) and a volcano plot (D). Red indicates higher mRNA expression levels and blue indicates lower expression in PAs versus non-PAs. Differentially expressed genes were identified using the EdgeR package with adjusted p-value cutoffs <0.05 and log2 fold changes > 2. **(E)** Independent validation of differential gene expression using mRNA isolated from PAs (EGFP^+^/DsRed^+^) and non-PAs (EGFP^-^/DsRed^+^). Note that the select genes analyzed show enrichment in PAs as compared to non-PAs, as revealed by qRT-PCR. Error bars indicate SE of the mean, n = 3. The red bars indicate EGFP^-^/DsRed^+^ cell fractions. Expression differences between sorted cells were determined using two-way analysis of variance with Bonferroni post-hoc test, *p<0.05, **p<0.01, ***p<0.001, and ****p<0.0001.

To isolate sub-populations of PAs (*EGFP*^+^/ *DsRed*^+^) and non-PAs (DsRed^+^) for gene expression comparisons, cerebral cortices from adult Mlc1-EGFP;GLAST-DsRed animals (n = 4) were dissociated. EGFP^+^/DsRed^+^ double positive cells (PAs) and DsRed^+^ single positive cells (non-PAs) were then fractionated by FACS (Fig 5A and 5B). Cortical cell suspensions prepared from Mlc1-EGFP or GLAST-DsRed single transgenic mice were used to control for gating by FACS (S4 Fig). Quantitative RNA sequencing comparisons (n = 4 mice) were performed using these two main cell populations. These data reveal that PAs express a unique set of genes that are not expressed at similar levels in the non-PA populations (Fig 5C and S5 Fig). Using this RNAseq data, we performed unsupervised gene set enrichment analysis for all the Kyoto Encyclopedia of Genes and Genes and Genomes (KEGG) pathways. We found that multiple signaling pathways were enriched in PAs, including ones involved in epithelial to mesenchymal transition, hedgehog signaling, and angiogenesis (S9 Table). Several differentially expressed genes identified in PAs encode proteins with links to extracellular matrix adhesion and signaling pathways. These genes include *Itga7*, *Lama3*, *Lama5*, *Frem1*, *and Frem2*. Additional genes were also identified with roles in cell-cell contact and communication including *Sema4a*, *Sema4b*, *Gdf10*, and *Gjb2* (Fig 5D and S10 Table). The 50 most highly enriched genes in the non-PA cell fractions (de-enriched in the PA fractions) are shown in S11 Table. A table containing numerical details, including p-values and average fold change for all genes analyzed in PAs versus non-PA fractions is also provided (S12 Table).

**Fig 6:**
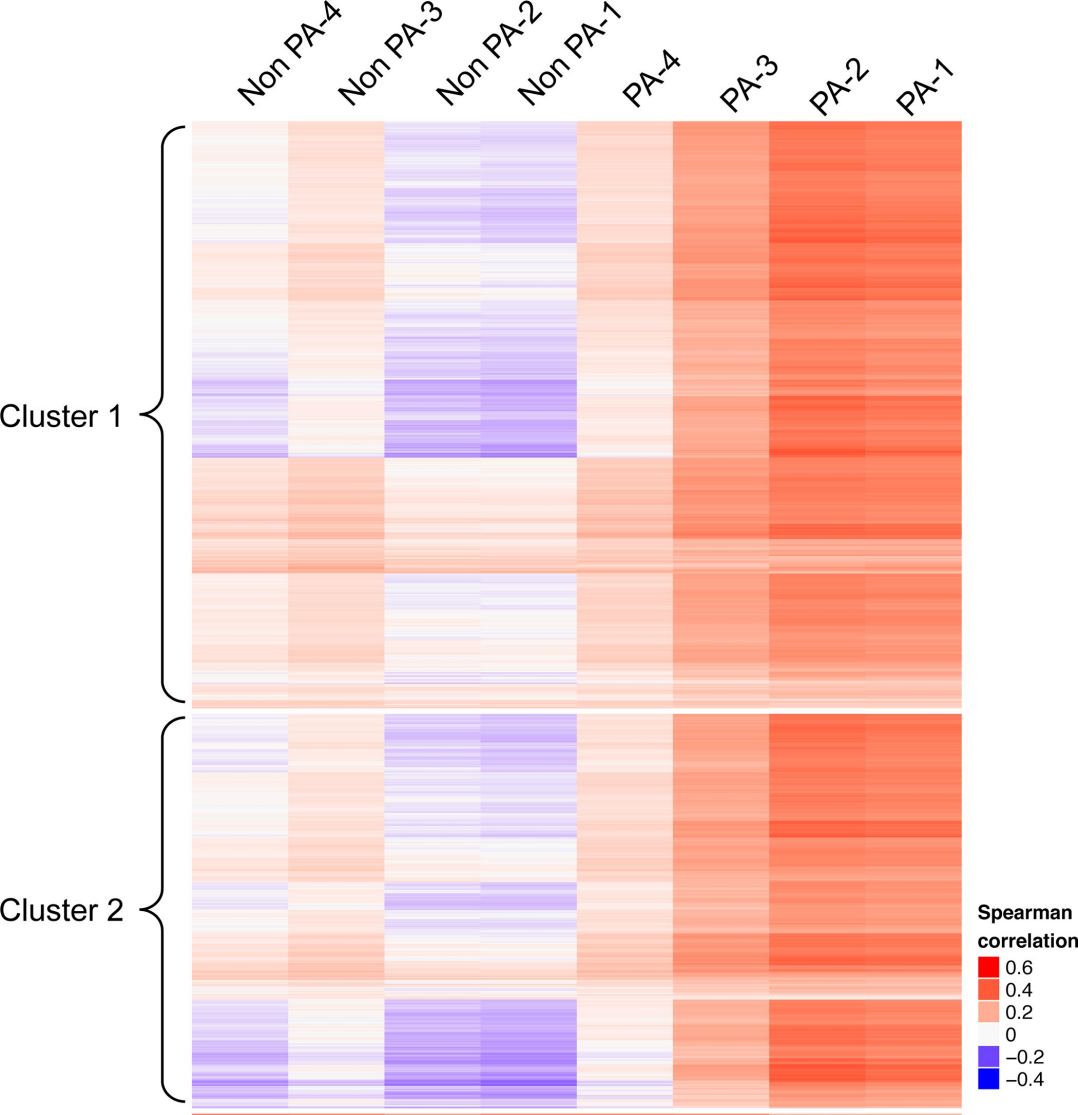
Correlation analyses of bulk RNAseq and scRNAseq data. Expression of genes in PAs (EGFP^+^) fractionated from Mlc1-EGFP mice and analyzed by single cell RNAseq (cell clusters 1 and 2, see Fig 2) were compared to gene expression patterns of PAs (EGFP^+^/DsRed^+^) and non-PAs (DsRed^+^) isolated from Mlc1-EGFP;GLAST-DsRed mice (n = 4). Note that gene expression patterns in purified PAs based on EGFP^+^ or EGFP^+^/DsRed^+^ expression correlate with each other, but do not correlate with non-PA expression patterns. Single cells from the scRNAseq analyses are shown along the y-axis. Heatmaps were colored using Pearson correlation coefficients between the gene expression of single cells in rows (y-axis) and expression of bulk samples (PA and non-PA samples) in columns (x-axis).

Consistent with the RNAseq data, qRT-PCR validation showed elevated levels of mRNAs in PAs as compared to non-PAs (Fig 5E). Correlation analyses between bulk RNAseq samples and scRNAseq data (clusters 1 and 2 in Fig 2) revealed that PAs (EGFP^+^/DsRed^+^) isolated from Mlc1-EGFP;GLAST-DsRed mice showed similar gene expression patterns with EGFP^+^ cells isolated from Mlc1-EGFP mice (Fig 6). Importantly, non-PAs did not segregate with the EGFP^+^ cells. Lastly, we compared 10 PA-enriched genes identified and validated from the bulk RNAseq data, as well as 5 canonical astrocyte genes, with a published transcriptome dataset describing five sub-populations of astrocytes fractionated from three different brain regions based on differential expression of various cell surface markers [18]. As shown in S6 Fig, we detect some overlap in the expression of the PA-enriched genes with sub-sets of fractionated astrocytes, further supporting the PA identities of the fractionated cells from the Mlc1-EGFP mouse model. In summary, these various data reveal that we have isolated and transcriptionally profiled a new sub-population of astrocytes with likely roles in regulating brain vascular functions.

## Discussion

In this report we have used genetically engineered mouse models, fluorescence-based cell fractionation, and quantitative RNA sequencing to identify several PA-expressed genes with putative links to vascular physiology. One central theme emerging from the data is that PA-enriched genes encode components linked to the ECM and cell adhesion. Prior studies of the brain vasculature revealed at least two different basement membranes (vascular endothelial and astrocyte) within the NVU [43]. PA-expressed ECM components identified from the RNAseq data include *Lama3*, which encodes the laminin α3 component of laminin-332 [44] as well as *Lama5* which encodes the α5 laminin subunit of laminin-511 [45]. These laminin subunits interact with other ECM proteins and have established roles in basement membrane biology in different organs. Loss-of-function mutations in human *LAMA3* gene are found in heritable forms of epidermolysis bullosa [46] and *Lama3* deletion in mice leads to similar skin blistering pathologies [47]. Laminin-511 in the vascular endothelial basement membrane is important for blood vessel integrity after experimentally-induced hemorrhagic stroke [48]. In this same study, roles for *Lama5* in BBB physiology in the healthy brain were not evident, possibly due to compensation by *Lama3* or other ECM proteins. It is possible that *Lama5* and *Lama3* expressed by PAs play key roles in NVU cytoarchitecture and BBB integrity under physiological settings. Interestingly, the RNAseq screen also identified the *Itga7* gene, which encodes α7 integrin protein that dimerizes exclusively with β1 integrin. The integrin α7β1 is a receptor for laminins [49], with deletion of *Itga7* in mice leading to muscular dystrophy-like phenotypes due to defective adhesion and signaling pathways in skeletal muscle cells [50]. Some *Itga7*-/- mice develop vascular pathologies including cranial hemorrhage, which has been attributed to loss of α7β1 functions in vascular smooth muscle cells [51]. Our results suggest that α7β1 integrin in PAs is contributing additional roles in the vasculature, possibly via adhesion to laminin protein ligands in the astrocyte basement membrane. It will be interesting to determine if ablation of *Lama5*/*Lama3* or the *Itga7* genes in PAs impacts BBB permeability in physiological or pathological contexts. Along these lines, we are currently developing an Mlc1-CreERT2 knock-in mouse model for tamoxifen-inducible deletion of genes selectively in PAs.

In addition to laminins, the RNAseq experiments identified other ECM factors with roles in basement membrane integrity, including *Frem1* and *Frem2*. Mutations in *FREM1* or *FREM2* often result in human birth defects including congenital diaphragmatic hernia as well as abnormalities in craniofacial and renal development [52, 53]. The cell surface receptors for the Frem proteins have not been definitively identified [54]. It will be interesting to determine if α7β1 integrin is a receptor for these ECM proteins and whether there are interactions between the Frem proteins and laminins. PAs also selectively express various collagens, including *Col16a1*, *Col11a2*, and *Col4a5* as well as *P4ha2*, which encodes the alpha-2 subunit of prolyl 4-hydroxylase, a key enzyme involved in collagen synthesis and ECM deposition [55]. These results bolster our model for PA basement membrane control of blood vessel stability and BBB homeostasis.

PAs also express *Sema4a* and *Sema4b*, both members of the Semaphorin gene family that encode counter-receptors for plexins, neuropilins and some growth factor receptor tyrosine kinases [56]. Semaphorin 4a and 4b also have short cytoplasmic domains with likely signaling functions [57]. Phosphorylation of the Semaphorin 4b cytoplasmic domain has been shown to play important roles in reactive astrogliosis, with *Sema4b*-/- mice showing defects in the migration of reactive astrocytes to damaged regions following experimental brain injury [58]. PA expression of intracellular enzymes such as the kinase Alkp1, suggest that cell-intrinsic signaling pathways may control communication between PAs and vascular cells. Along these lines, genetic deletion of *Alkp1* in mice leads to neurodegenerative phenotypes that may be due to BBB permeability defects [59]. Lastly, Mical-l2/Jrab is a PA-enriched actin cytoskeleton adapter protein that promotes cell-cell junction formation in epithelial cells via transmembrane protein recycling through Rab GTPases [60, 61]. PAs also express *Als2cl*, which encodes a Rab GTPase exchange factor, suggesting that regulation of Rab activities via the Mical-l2/Alsin pathway may control cell surface protein expression important for PA adhesion to the ECM.

The TGFβ superfamily member, *Gdf10*, was identified as a PA-enriched gene product. While glial-derived TGFβ signaling is important for angiogenesis and BBB formation during development [62], it remains uncertain if canonical TGFβ signaling in adult brain ECs is critical for BBB physiology. *Gdf10* is also expressed by adult brain ECs and promotes astrocyte and neuronal survival as well as BBB repair following stroke [63]. It will be important to determine if PA-derived Gfd10 protein is also playing a major role in blood vessel stability and BBB homeostasis in the healthy brain. Interestingly, we also identified *Prelp* as a gene enriched in PAs. *Prelp* encodes prolargin, a proline/arginine-rich end leucine-rich repeat protein and member of leucine rich repeat family of proteins that are expressed in ECM. Prior studies have shown that the related leucine rich repeat containing protein 33 is secreted into the brain microenvironment by microglia and promotes latent-TGFβ activation and signaling [64]. Hence, it will be important to determine if prolargin interacts with Gdf10 or other TGFβ family members to regulate PA adhesion and signaling functions. GSEA shows a Notch pathway signature involving several PA-expressed genes linked to the control of Notch processing, activation and transcriptional regulation. For example, *Lnfg* encodes a glycosyltransferase involved in Notch post-translational modification [65]. *Maml2* encodes mastermind 1, a Notch transcriptional regulatory factor [66]. How Notch signaling components may intersect with these events, as has been reported previously in studies of CNS angiogenesis [67], will be important to analyze. Links between these pathway components and Hedgehog signaling, which we also identified by GSEA as a signature pathway in PAs and has been reported previously to regulate BBB integrity and neuroinflammation [22], will also be interesting to study.

Abnormal control of BBB permeability is linked to the pathogenesis of various neurological diseases [68]. Many of the genes and pathways that are deregulated and contribute to neurovascular disease onset and progression remain largely unknown. It will be important to determine if deregulation of any of the PA-expressed genes we have identified in this study are altered in neurovascular-related brain disorders. Since mutations in *MLC1* cause the neurodevelopmental disorder MLC [69], it is possible that alterations in additional PA-expressed genes are linked to BBB defects in other pathologies. Lastly, there are human brain diseases, including various psychiatric disorders and age-related neurodegenerative pathologies, that develop without overt changes in BBB permeability. In these pathologies, the highly selective permeability properties of the BBB makes it a major impediment for delivering therapeutics [70]. Hence, there is a critical need to identify pathways that can be manipulated to allow reliable and effective delivery of drugs to the brain. Studying the PA-expressed genes identified in this study; therefore, will not only be informative for understanding the basic biology and pathophysiology of the NVU, but may also be potential targets for selectively modulating BBB permeability to enhance drug delivery to treat neurological diseases.

## Supporting information

S1 Fig Single cell RNAseq analysis in PAs isolated from adult Mlc1-EGFP mice. Quantitative expression comparisons between the 7 different cell clusters identified by scRNAseq. Red indicates higher mRNA expression levels and blue indicates lower expression in EGFP-expressing PAs. The differentially expressed genes were identified using the SEURAT package.
(TIF)

S2 Fig Unbiased differential gene expression comparisons of PA clusters 1 and 2. Unbiased expression comparisons of cell clusters 1 and 2 from scRNAseq reveal various differentially expressed genes. These data are based on adjusted p-value cutoff < 0.01 (Wilcoxon rank-sum test) and log2 fold change > 1.
(TIF)

S3 Fig Comparative analysis of defined vascular and neural cell markers in isolated PAs. Feature plots showing the expression of select mRNAs with established roles in vascular endothelial cells (*Cldn5* and *Pecam1*), pericytes (*Des* and *Acta2*), microglia (Il*1b* and *Tgf1*), neurons (*Trb1* and *Tubb3*), oligodendrocyte progenitor cells (*Pdgfra* and *Myrf*), and astroglial cells of the neurovascular unit (*Itgb8* and *Vegfa*) plotted by UMAP. Each dot indicates an RNA sequencing result from a different analyzed cell. Red indicates higher gene expression and grey indicates lower gene expression. Note that cell clusters one and two mainly show enrichment for the *Itgb8* and *Vegfa* mRNAs. The five other cell clusters do not show significant enrichment in the non-neurovascular markers.
(TIF)

S4 Fig Gating controls for isolation of PAs from the adult mouse brain by FACS. **(A-C);** Cerebral cortical cell suspensions from Mlc1-EGFP mice (A), GLAST-DsRed mice (B) or Mlc1-EGFP/GLAST-DsRed double-positive mice (C) were used for fractionation of EGFP^+^ single positive or EGFP^+^/DsRed^+^ double positive cells PAs or DsRed^+^ single positive non-PAs. Single positive cells in panels A and B were used as gating controls for the double positive cell fractionation shown in panel C.
(TIF)

S5 Fig Quantitative RNA sequencing to identify differentially expressed genes in PAs versus non-PAs isolated from Mlc1-EGFP;GLAST-DsRed adult mice. Shown is a complete list of differentially expressed genes in PAs versus non-PAs as revealed by a color-coded heat map. The heat map includes the same samples shown in Fig 5C, but with all differentially expressed genes identified along the y-axis. The differentially expressed genes were identified using the EdgeR package with an adjusted p-value cutoff 0.05 and log2 fold change > 2.
(TIF)

S6 Fig Cross-referencing a 15 gene signature with published astrocyte transcriptome datasets. Ten genes with enriched expression in PAs, and five canonical astrocyte genes (*Mlc1*, *Slc1a3*, *Gfap*, *Aldh1l1*, and *Itgb8*), were compared by hierarchical clustering to a published bulk RNAseq report (PMC5824716) involving five astrocyte sub-populations (termed A-E) isolated from three different brain regions (olfactory bulb, region 1; cortex, region 2; and brain stem, region 3). Note the partial overlap in expression between the PA-enriched genes and different astrocyte sub-populations in the published study. The individual genes in the 15 gene signature are shown in rows (y-axis) and the various sub-sets of astrocytes from different brain regions are shown in columns (x-axis).
(TIF)

S1 Table Summary of commercial antibodies. (DOCX)

S2 Table The 20 most enriched genes in cell cluster 1 from scRNAseq. (DOCX)

S3 Table The 20 most enriched genes in cell cluster 2 from scRNAseq. (DOCX)

S4 Table The 20 most enriched genes in cell cluster 3 from scRNAseq. (DOCX)

S5 Table The 20 most enriched genes in cell cluster 4 from scRNAseq. (DOCX)

S6 Table The 20 most enriched genes in cell cluster 5 from scRNAseq. (DOCX)

S7 Table The 20 most enriched genes in cell cluster 6 from scRNAseq. (DOCX)

S8 Table The 20 most enriched genes in cell cluster 7 from scRNAseq. (DOCX)

S9 Table The top 10 signaling pathways in PAs as determined by Gene Set Enrichment Analyses (GSEA). (DOCX)

S10 Table The top 30 genes with enriched expression in PAs versus non-PAs based on bulk RNAseq. (DOCX)

S11 Table (XLSX)

S12 Table (XLSX)
