# Supplementary figures and images for: Isolation and transcriptional characterization of mouse perivascular astrocytes
Source: PLoS One. 2020 Oct 8;15(10):e0240035. doi: 10.1371/journal.pone.0240035 (PMC7544046; doi:10.1371/journal.pone.0240035)

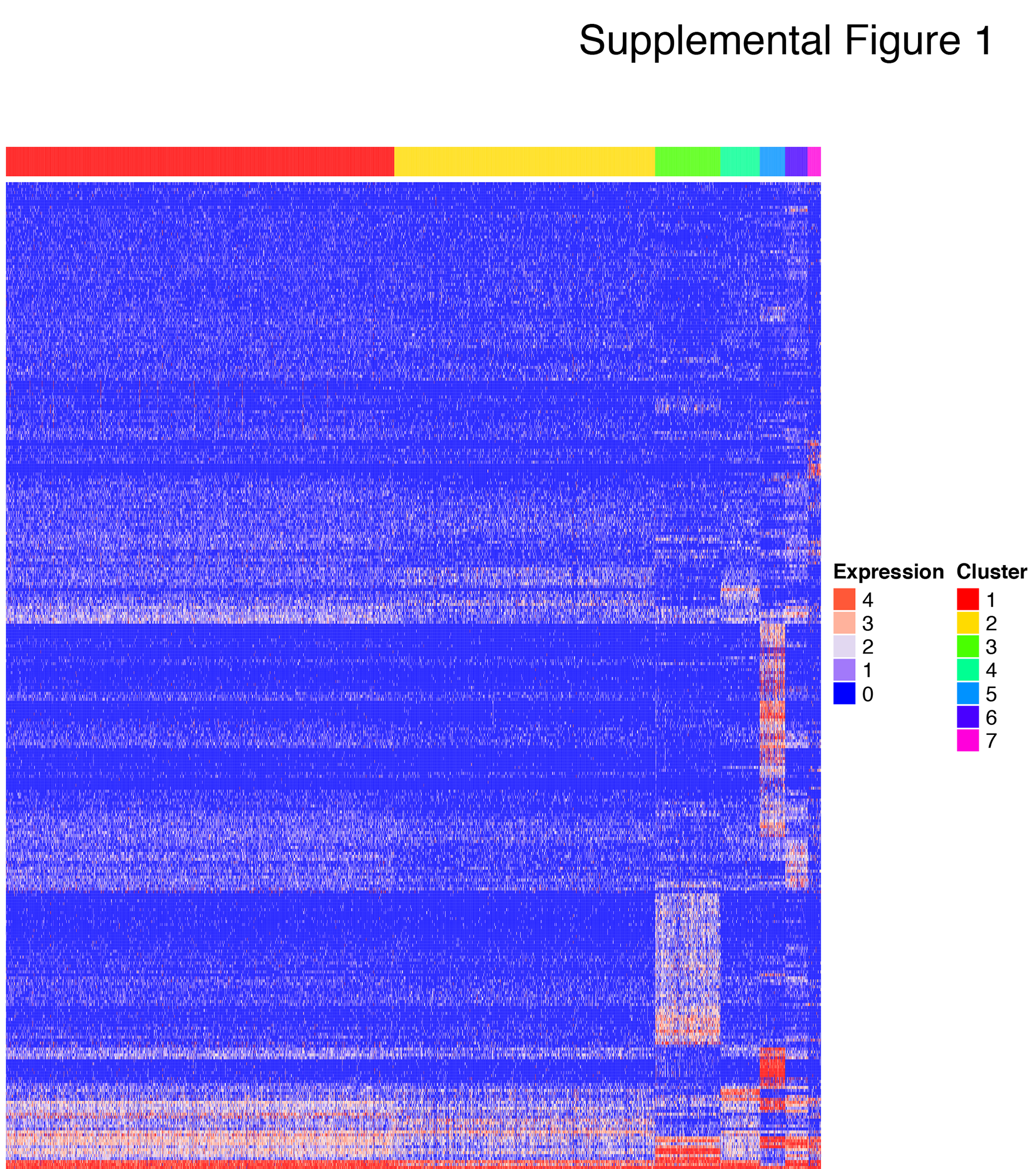

Supplement: S1 Fig — Quantitative expression comparisons between the 7 different cell clusters identified by scRNAseq. Red indicates higher mRNA expression levels and blue indicates lower expression in EGFP-expressing PAs. The differentially expressed genes were identified using the SEURAT package. (TIF) [file pone.0240035.s001.tif]

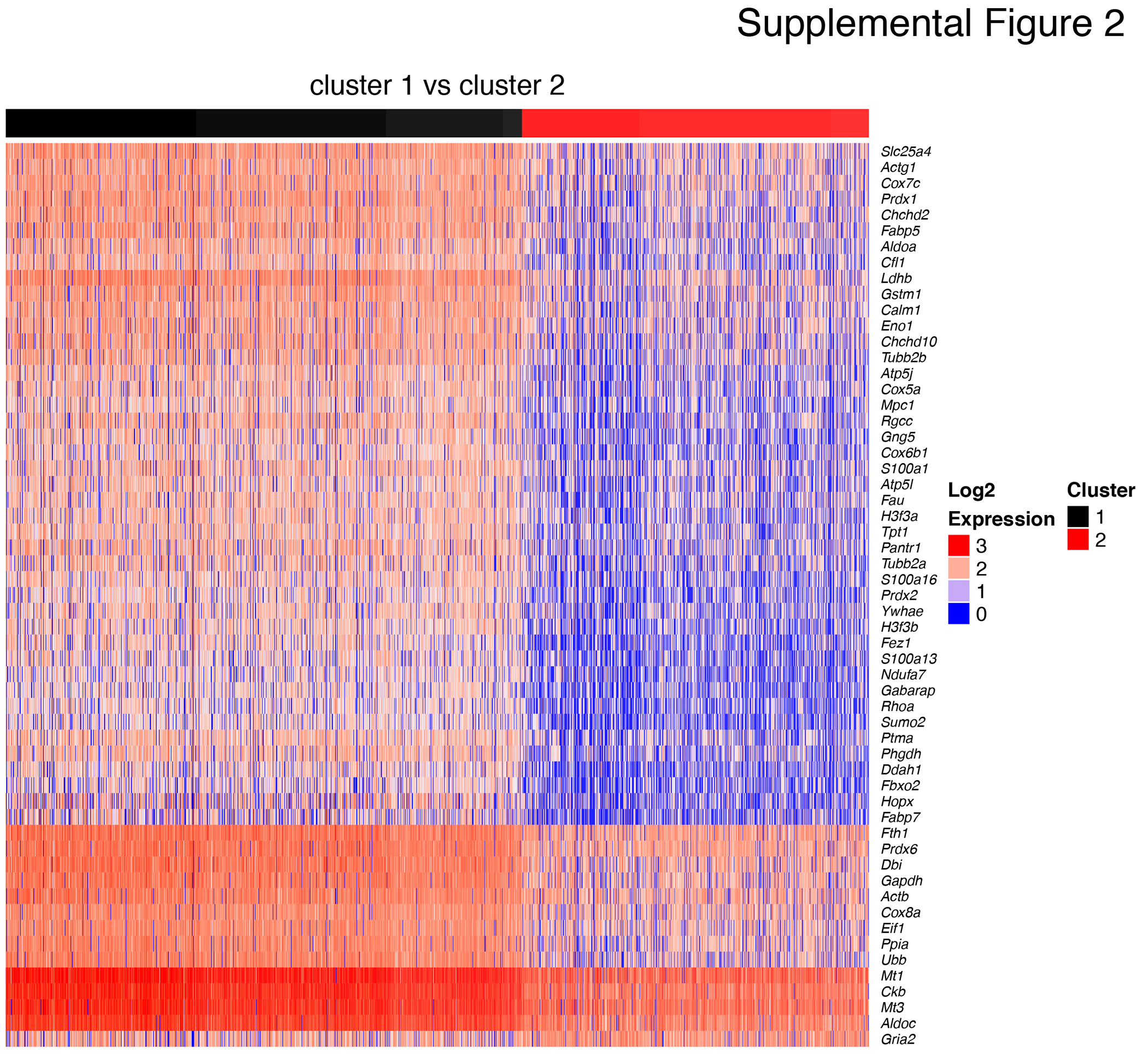

Supplement: S2 Fig — Unbiased expression comparisons of cell clusters 1 and 2 from scRNAseq reveal various differentially expressed genes. These data are based on adjusted p-value cutoff < 0.01 (Wilcoxon rank-sum test) and log2 fold change > 1. (TIF) [file pone.0240035.s002.tif]

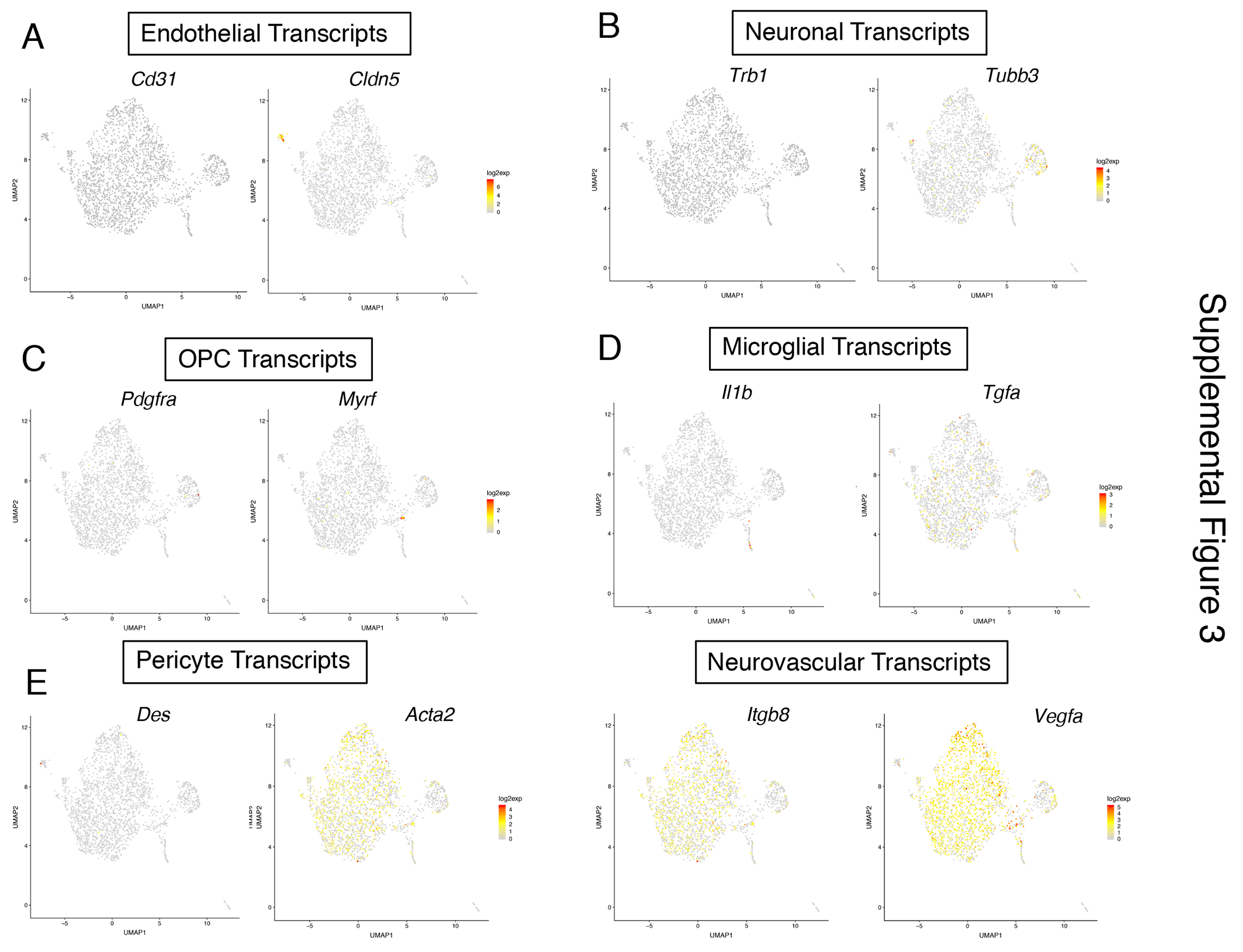

Supplement: S3 Fig — Feature plots showing the expression of select mRNAs with established roles in vascular endothelial cells (Cldn5 and Pecam1), pericytes (Des and Acta2), microglia (Il1b and Tgf1), neurons (Trb1 and Tubb3), oligodendrocyte progenitor cells (Pdgfra and Myrf), and astroglial cells of the neurovascular unit (Itgb8 and Vegfa) plotted by UMAP. Each dot indicates an RNA sequencing result from a different analyzed cell. Red indicates higher gene expression and grey indicates lower gene expression. Note that cell clusters one and two mainly show enrichment for the Itgb8 and Vegfa mRNAs. The five other cell clusters do not show significant enrichment in the non-neurovascular markers. (TIF) [file pone.0240035.s003.tif]

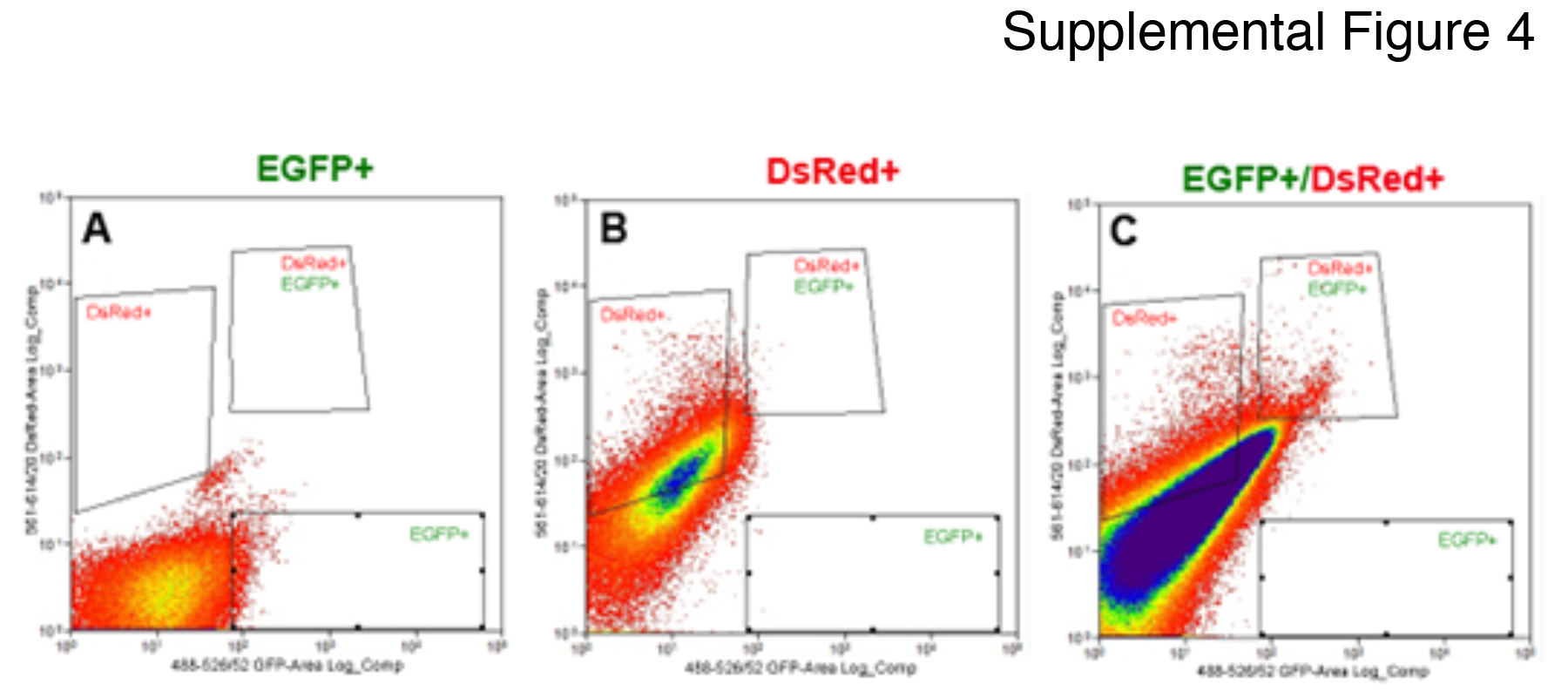

Supplement: S4 Fig — (A-C); Cerebral cortical cell suspensions from Mlc1-EGFP mice (A), GLAST-DsRed mice (B) or Mlc1-EGFP/GLAST-DsRed double-positive mice (C) were used for fractionation of EGFP+ single positive or EGFP+/DsRed+ double positive cells PAs or DsRed+ single positive non-PAs. Single positive cells in panels A and B were used as gating controls for the double positive cell fractionation shown in panel C. (TIF) [file pone.0240035.s004.tif]

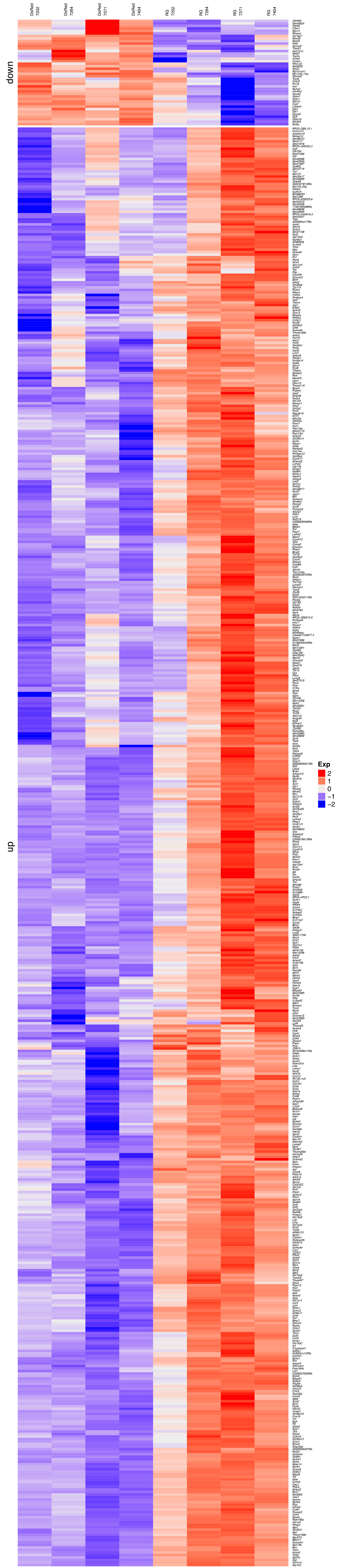

Supplement: S5 Fig — Shown is a complete list of differentially expressed genes in PAs versus non-PAs as revealed by a color-coded heat map. The heat map includes the same samples shown in Fig 5C, but with all differentially expressed genes identified along the y-axis. The differentially expressed genes were identified using the EdgeR package with an adjusted p-value cutoff 0.05 and log2 fold change > 2. (TIF) [file pone.0240035.s005.tif]

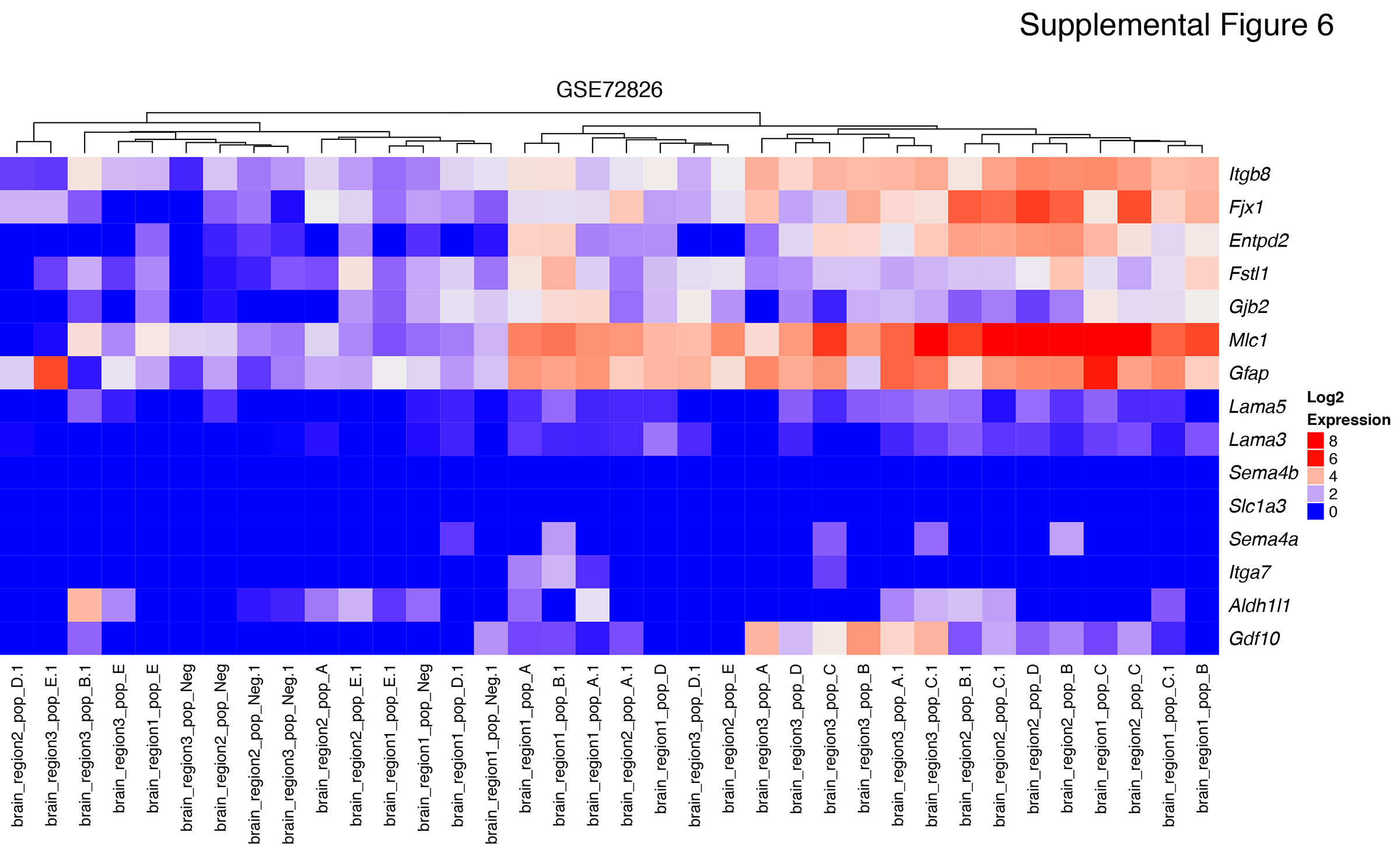

Supplement: S6 Fig — Ten genes with enriched expression in PAs, and five canonical astrocyte genes (Mlc1, Slc1a3, Gfap, Aldh1l1, and Itgb8), were compared by hierarchical clustering to a published bulk RNAseq report (PMC5824716) involving five astrocyte sub-populations (termed A-E) isolated from three different brain regions (olfactory bulb, region 1; cortex, region 2; and brain stem, region 3). Note the partial overlap in expression between the PA-enriched genes and different astrocyte sub-populations in the published study. The individual genes in the 15 gene signature are shown in rows (y-axis) and the various sub-sets of astrocytes from different brain regions are shown in columns (x-axis). (TIF) [file pone.0240035.s006.tif]
