# Supplementary material for: Isolation and transcriptional characterization of mouse perivascular astrocytes
Source: PLoS One. 2020 Oct 8;15(10):e0240035. doi: 10.1371/journal.pone.0240035 (PMC7544046; doi:10.1371/journal.pone.0240035)
Supplement: S1 Table — (DOCX) [file pone.0240035.s007.docx]

**S1 Table. Summary of commercial antibodies.**

| **Primary Antibody** | **Application/Dilution** | **Cat. number** | **Company** |
| --- | --- | --- | --- |
| Chicken anti-GFP | IF (1:500) | GFP-1020 | Aves Labs |
| Rabbit anti-RFP | IF (1:200) | AB62341 | Abcam |
| Mouse anti-GFAP | IF (1:200) | MAB3402 | EMD-Millipore |
| Rat anti-CD31 | IF (1:200) | 553370 | BD Pharmingen |

| **Secondary Antibody** | **Application** | **Cat. number** | **Company** |
| --- | --- | --- | --- |
| Alexa Flour® 488 Donkey anti-Chicken | IF for GFP | 703-545-155 | Jackson ImmunoResearch |
| Alexa Flour® 568 Donkey anti-Rabbit | IF for RFP | A10042 | Life Technologies |
| Alexa Flour® 647 Donkey anti-Mouse | IF for GFAP | 715-605-151 | Jackson ImmunoResearch |
| Alexa Flour® 647 Donkey anti-Rat | IF for CD31 | 712-605-153 | Jackson ImmunoResearch |
