# Supplementary material for: Isolation and transcriptional characterization of mouse perivascular astrocytes
Source: PLoS One. 2020 Oct 8;15(10):e0240035. doi: 10.1371/journal.pone.0240035 (PMC7544046; doi:10.1371/journal.pone.0240035)
Supplement: S2 Table — (DOCX) [file pone.0240035.s008.docx]

**S2 Table. The 20 most enriched genes in cell cluster 1 from scRNAseq.**

| **Gene** | **p_val** | **avg_logFC** | **pct.1** | **pct.2** | **p_val_adj** | **cluster** |
| --- | --- | --- | --- | --- | --- | --- |
| *Aldoc* | 1.99E-191 | 0.77409 | 0.995 | 0.938 | 3.67E-187 | 1 |
| *Ckb* | 1.01E-171 | 0.673864 | 1 | 0.979 | 1.87E-167 | 1 |
| *Prdx6* | 3.69E-168 | 0.728329 | 0.979 | 0.884 | 6.81E-164 | 1 |
| *Mt1* | 7.23E-156 | 0.665413 | 0.999 | 0.981 | 1.33E-151 | 1 |
| *Rgcc* | 2.88E-153 | 0.88804 | 0.934 | 0.573 | 5.31E-149 | 1 |
| *Ldhb* | 1.33E-150 | 0.722818 | 0.971 | 0.829 | 2.45E-146 | 1 |
| *Mt3* | 1.01E-141 | 0.586212 | 0.993 | 0.955 | 1.86E-137 | 1 |
| *Prdx1* | 7.45E-141 | 0.701857 | 0.96 | 0.778 | 1.38E-136 | 1 |
| *Glul* | 2.55E-137 | 0.577383 | 0.987 | 0.951 | 4.70E-133 | 1 |
| *Gapdh* | 3.08E-136 | 0.653317 | 0.977 | 0.876 | 5.69E-132 | 1 |
| *Fez1* | 1.13E-117 | 0.729657 | 0.892 | 0.496 | 2.08E-113 | 1 |
| *Phgdh* | 3.59E-117 | 0.706745 | 0.921 | 0.598 | 6.63E-113 | 1 |
| *Pantr1* | 1.30E-111 | 0.746433 | 0.925 | 0.663 | 2.39E-107 | 1 |
| *Fabp5* | 7.86E-110 | 0.718844 | 0.951 | 0.723 | 1.45E-105 | 1 |
| *Gstm5* | 3.03E-102 | 0.608727 | 0.958 | 0.827 | 5.59E-98 | 1 |
| *Eif1* | 5.75E-101 | 0.502025 | 0.973 | 0.869 | 1.06E-96 | 1 |
| *S100a16* | 2.32E-99 | 0.616112 | 0.925 | 0.599 | 4.27E-95 | 1 |
| *Ubb* | 2.77E-98 | 0.494353 | 0.976 | 0.843 | 5.11E-94 | 1 |
| *Aldoa* | 2.87E-98 | 0.570354 | 0.947 | 0.745 | 5.29E-94 | 1 |
| *Hopx* | 3.00E-96 | 1.013102 | 0.822 | 0.467 | 5.54E-92 | 1 |
