# Supplementary material for: Isolation and transcriptional characterization of mouse perivascular astrocytes
Source: PLoS One. 2020 Oct 8;15(10):e0240035. doi: 10.1371/journal.pone.0240035 (PMC7544046; doi:10.1371/journal.pone.0240035)
Supplement: S3 Table — (DOCX) [file pone.0240035.s009.docx]

**S3 Table. The 20 most enriched genes in cell cluster 2 from scRNAseq.**

| **Gene** | **p_val** | **avg_logFC** | **pct.1** | **pct.2** | **p_val_adj** | **cluster** |
| --- | --- | --- | --- | --- | --- | --- |
| *Ubb* | 2.03E-163 | -0.93556 | 0.765 | 0.973 | 3.75E-159 | 2 |
| *Ppia* | 1.44E-133 | -0.75829 | 0.809 | 0.967 | 2.66E-129 | 2 |
| *Fth1* | 1.56E-120 | -0.95584 | 0.925 | 0.995 | 2.88E-116 | 2 |
| *Chchd2* | 1.16E-119 | -0.7309 | 0.733 | 0.955 | 2.15E-115 | 2 |
| *Gapdh* | 3.66E-117 | -0.72638 | 0.841 | 0.963 | 6.76E-113 | 2 |
| *Tpt1* | 5.32E-117 | -1.02683 | 0.666 | 0.939 | 9.81E-113 | 2 |
| *Atp1a2* | 9.51E-117 | 0.671259 | 0.992 | 0.922 | 1.76E-112 | 2 |
| *Malat1* | 1.18E-111 | 0.671128 | 0.999 | 0.995 | 2.18E-107 | 2 |
| *Eif1* | 2.09E-111 | -0.64229 | 0.831 | 0.96 | 3.86E-107 | 2 |
| *Rpl41* | 6.92E-111 | -1.04957 | 0.784 | 0.96 | 1.28E-106 | 2 |
| *Dbi* | 8.02E-111 | -0.82728 | 0.856 | 0.969 | 1.48E-106 | 2 |
| *Gria2* | 1.34E-108 | 0.843064 | 0.94 | 0.748 | 2.47E-104 | 2 |
| *Rps10* | 5.31E-107 | -0.92862 | 0.605 | 0.914 | 9.81E-103 | 2 |
| *Rps8* | 2.45E-106 | -0.98296 | 0.691 | 0.949 | 4.51E-102 | 2 |
| *Ckb* | 2.57E-105 | -0.62552 | 0.981 | 0.993 | 4.75E-101 | 2 |
| *Rpl11* | 5.52E-104 | -0.89768 | 0.564 | 0.911 | 1.02E-99 | 2 |
| *Rpl15* | 6.55E-103 | -0.83682 | 0.533 | 0.881 | 1.21E-98 | 2 |
| *Ldhb* | 5.47E-102 | -0.70011 | 0.803 | 0.941 | 1.01E-97 | 2 |
| *Rps27a* | 3.87E-101 | -1.01489 | 0.605 | 0.918 | 7.14E-97 | 2 |
| *Son* | 2.79E-100 | 0.648915 | 0.976 | 0.856 | 5.16E-96 | 2 |
