# Supplementary material for: Isolation and transcriptional characterization of mouse perivascular astrocytes
Source: PLoS One. 2020 Oct 8;15(10):e0240035. doi: 10.1371/journal.pone.0240035 (PMC7544046; doi:10.1371/journal.pone.0240035)
Supplement: S4 Table — (DOCX) [file pone.0240035.s010.docx]

**S4 Table. The 20 most enriched genes in cell cluster 3 from scRNAseq.**

| **Gene** | **p_val** | **avg_logFC** | **pct.1** | **pct.2** | **p_val_adj** | **cluster** |
| --- | --- | --- | --- | --- | --- | --- |
| *Camk2a* | 3.72E-244 | 2.177706 | 0.901 | 0.067 | 6.87E-240 | 3 |
| *Gng13* | 1.41E-242 | 1.64269 | 0.796 | 0.04 | 2.59E-238 | 3 |
| *Nrsn1* | 8.26E-233 | 1.96935 | 0.845 | 0.058 | 1.53E-228 | 3 |
| *Ddn* | 9.10E-230 | 1.232007 | 0.591 | 0.011 | 1.68E-225 | 3 |
| *Psd* | 1.61E-229 | 1.936931 | 0.829 | 0.056 | 2.98E-225 | 3 |
| *Hpcal4* | 1.17E-223 | 1.286873 | 0.619 | 0.016 | 2.17E-219 | 3 |
| *Trnp1* | 1.09E-215 | 1.293705 | 0.608 | 0.017 | 2.01E-211 | 3 |
| *Shank1* | 2.12E-215 | 1.52496 | 0.669 | 0.027 | 3.92E-211 | 3 |
| *Cnih2* | 2.10E-209 | 1.728554 | 0.768 | 0.051 | 3.87E-205 | 3 |
| *Nrgn* | 4.19E-204 | 2.403392 | 0.912 | 0.104 | 7.74E-200 | 3 |
| *Kif5a* | 1.57E-196 | 1.842449 | 0.845 | 0.081 | 2.91E-192 | 3 |
| *Dlgap3* | 2.36E-188 | 0.954399 | 0.508 | 0.011 | 4.35E-184 | 3 |
| *Nefl* | 7.09E-188 | 1.237532 | 0.558 | 0.018 | 1.31E-183 | 3 |
| *Eef1a2* | 1.06E-185 | 1.153989 | 0.602 | 0.026 | 1.95E-181 | 3 |
| *Bc1* | 2.62E-185 | 1.020608 | 0.541 | 0.017 | 4.83E-181 | 3 |
| *Sptbn2* | 5.83E-182 | 0.977769 | 0.47 | 0.008 | 1.08E-177 | 3 |
| *Zfp365* | 7.66E-174 | 1.31184 | 0.669 | 0.046 | 1.41E-169 | 3 |
| *Celf4* | 2.14E-173 | 1.548943 | 0.729 | 0.063 | 3.96E-169 | 3 |
| *Cck* | 6.21E-173 | 1.315839 | 0.541 | 0.021 | 1.15E-168 | 3 |
| *Ppp2r2c* | 1.69E-169 | 1.02331 | 0.558 | 0.025 | 3.13E-165 | 3 |
