# Supplementary material for: Isolation and transcriptional characterization of mouse perivascular astrocytes
Source: PLoS One. 2020 Oct 8;15(10):e0240035. doi: 10.1371/journal.pone.0240035 (PMC7544046; doi:10.1371/journal.pone.0240035)
Supplement: S5 Table — (DOCX) [file pone.0240035.s011.docx]

**S5 Table. The 20 most enriched genes in cell cluster 4 from scRNAseq.**

| **Gene** | **p_val** | **avg_logFC** | **pct.1** | **pct.2** | **p_val_adj** | **cluster** |
| --- | --- | --- | --- | --- | --- | --- |
| *Nkx6-2* | 4.85E-207 | 2.223629 | 0.826 | 0.039 | 8.95E-203 | 4 |
| *Igsf1* | 1.24E-150 | 1.153317 | 0.578 | 0.024 | 2.28E-146 | 4 |
| *Sfrp5* | 9.13E-93 | 0.757719 | 0.45 | 0.027 | 1.69E-88 | 4 |
| *B230323A14Rik* | 8.04E-84 | 0.400639 | 0.183 | 0 | 1.48E-79 | 4 |
| *Agt* | 9.87E-82 | 2.203143 | 0.972 | 0.322 | 1.82E-77 | 4 |
| *Gria1* | 2.38E-69 | 0.859239 | 0.56 | 0.069 | 4.39E-65 | 4 |
| *Hotairm1* | 2.25E-65 | 0.317275 | 0.174 | 0.002 | 4.15E-61 | 4 |
| *Itih3* | 2.52E-62 | 1.956273 | 0.972 | 0.485 | 4.65E-58 | 4 |
| *Sparc* | 3.50E-55 | 1.431375 | 0.963 | 0.463 | 6.46E-51 | 4 |
| *Slc6a11* | 3.35E-53 | 1.38647 | 0.991 | 0.735 | 6.19E-49 | 4 |
| *Slc6a9* | 1.82E-45 | 0.921393 | 0.67 | 0.172 | 3.35E-41 | 4 |
| *Ucp2* | 8.58E-43 | 0.526186 | 0.404 | 0.055 | 1.58E-38 | 4 |
| *Ednrb* | 1.90E-42 | 1.168176 | 0.972 | 0.715 | 3.51E-38 | 4 |
| *Lrig1* | 3.10E-42 | 0.859124 | 0.734 | 0.217 | 5.72E-38 | 4 |
| *Spon1* | 1.79E-40 | 0.879608 | 0.697 | 0.209 | 3.30E-36 | 4 |
| *Gpld1* | 1.11E-38 | 1.074897 | 0.881 | 0.463 | 2.04E-34 | 4 |
| *Pipox* | 1.59E-34 | 0.621431 | 0.486 | 0.109 | 2.94E-30 | 4 |
| *B3galt2* | 1.32E-33 | 0.942183 | 0.752 | 0.288 | 2.44E-29 | 4 |
| *Paqr6* | 1.67E-33 | 0.825677 | 0.716 | 0.256 | 3.09E-29 | 4 |
| *Etnppl* | 2.20E-32 | 0.833349 | 0.716 | 0.25 | 4.06E-28 | 4 |
