# Supplementary material for: Isolation and transcriptional characterization of mouse perivascular astrocytes
Source: PLoS One. 2020 Oct 8;15(10):e0240035. doi: 10.1371/journal.pone.0240035 (PMC7544046; doi:10.1371/journal.pone.0240035)
Supplement: S6 Table — (DOCX) [file pone.0240035.s012.docx]

**S6 Table. The 20 most enriched genes in cell cluster 5 from scRNAseq.**

| **Gene** | **p_val** | **avg_logFC** | **pct.1** | **pct.2** | **p_val_adj** | **cluster** |
| --- | --- | --- | --- | --- | --- | --- |
| *Csf1r* | 0 | 3.092087 | 0.914 | 0.014 | 0 | 5 |
| *Laptm5* | 0 | 2.557697 | 0.943 | 0.01 | 0 | 5 |
| *Fcgr3* | 0 | 2.093437 | 0.829 | 0.008 | 0 | 5 |
| *Pld4* | 0 | 1.943089 | 0.814 | 0.002 | 0 | 5 |
| *Cd300c2* | 0 | 1.868458 | 0.8 | 0.005 | 0 | 5 |
| *Cd53* | 0 | 1.664382 | 0.843 | 0.005 | 0 | 5 |
| *Cfh* | 0 | 1.608814 | 0.8 | 0.005 | 0 | 5 |
| *Spi1* | 0 | 1.570647 | 0.8 | 0.006 | 0 | 5 |
| *Adgre1* | 0 | 1.493885 | 0.7 | 0.002 | 0 | 5 |
| *Fcgr1* | 0 | 1.224973 | 0.714 | 0.003 | 0 | 5 |
| *Alox5ap* | 1.55E-304 | 1.373525 | 0.7 | 0.003 | 2.86E-300 | 5 |
| *Ms4a6d* | 4.48E-300 | 1.258235 | 0.643 | 0.001 | 8.28E-296 | 5 |
| *Ly86* | 6.49E-299 | 2.674955 | 0.914 | 0.015 | 1.20E-294 | 5 |
| *Cd86* | 3.21E-294 | 1.057533 | 0.657 | 0.002 | 5.91E-290 | 5 |
| *Ltc4s* | 7.26E-294 | 1.890194 | 0.786 | 0.008 | 1.34E-289 | 5 |
| *Cd37* | 4.32E-289 | 1.145176 | 0.657 | 0.002 | 7.98E-285 | 5 |
| *Ptprc* | 1.75E-286 | 1.134815 | 0.629 | 0.001 | 3.22E-282 | 5 |
| *Ncf2* | 7.95E-286 | 0.915729 | 0.614 | 0.001 | 1.47E-281 | 5 |
| *Hpgds* | 2.40E-285 | 1.778074 | 0.814 | 0.011 | 4.43E-281 | 5 |
| *Hpgd* | 1.80E-284 | 1.893648 | 0.757 | 0.007 | 3.32E-280 | 5 |
