# Supplementary material for: Isolation and transcriptional characterization of mouse perivascular astrocytes
Source: PLoS One. 2020 Oct 8;15(10):e0240035. doi: 10.1371/journal.pone.0240035 (PMC7544046; doi:10.1371/journal.pone.0240035)
Supplement: S7 Table — (DOCX) [file pone.0240035.s013.docx]

**S7 Table. The 20 most enriched genes in cell cluster 6 from scRNAseq.**

| **Gene** | **p_val** | **avg_logFC** | **pct.1** | **pct.2** | **p_val_adj** | **cluster** |
| --- | --- | --- | --- | --- | --- | --- |
| *Mycn* | 1.08E-183 | 0.788894 | 0.435 | 0.002 | 1.99E-179 | 6 |
| *Bgn* | 1.11E-119 | 0.409106 | 0.371 | 0.005 | 2.05E-115 | 6 |
| *Draxin* | 5.01E-117 | 0.32665 | 0.306 | 0.002 | 9.25E-113 | 6 |
| *1500015O10Rik* | 1.08E-114 | 1.07901 | 0.403 | 0.008 | 1.99E-110 | 6 |
| *Ascl1* | 7.11E-108 | 1.878749 | 0.677 | 0.039 | 1.31E-103 | 6 |
| *Fxyd6* | 1.90E-107 | 0.949098 | 0.548 | 0.021 | 3.50E-103 | 6 |
| *Mfap2* | 6.05E-102 | 1.076842 | 0.694 | 0.044 | 1.12E-97 | 6 |
| *Insm1* | 4.32E-95 | 0.465547 | 0.258 | 0.002 | 7.98E-91 | 6 |
| *Bex4* | 5.34E-95 | 0.752564 | 0.629 | 0.036 | 9.85E-91 | 6 |
| *Pbk* | 5.12E-87 | 0.574758 | 0.177 | 0 | 9.46E-83 | 6 |
| *Mlf1* | 8.21E-83 | 0.480429 | 0.21 | 0.001 | 1.52E-78 | 6 |
| *Enkur* | 1.18E-82 | 0.64324 | 0.258 | 0.004 | 2.18E-78 | 6 |
| *Tox3* | 2.63E-82 | 0.896813 | 0.694 | 0.057 | 4.86E-78 | 6 |
| *Fbln2* | 8.84E-81 | 0.299274 | 0.355 | 0.01 | 1.63E-76 | 6 |
| *Fam174b* | 2.10E-77 | 0.270464 | 0.29 | 0.006 | 3.88E-73 | 6 |
| *Mn1* | 9.85E-77 | 0.401041 | 0.452 | 0.021 | 1.82E-72 | 6 |
| *Lockd* | 2.96E-75 | 0.479705 | 0.194 | 0.001 | 5.45E-71 | 6 |
| *Tmem132b* | 2.35E-74 | 0.489922 | 0.435 | 0.021 | 4.34E-70 | 6 |
| *Cd24a* | 2.04E-73 | 0.664942 | 0.21 | 0.002 | 3.76E-69 | 6 |
| *Thbs4* | 2.40E-73 | 0.777347 | 0.435 | 0.021 | 4.44E-69 | 6 |
