# Supplementary material for: Isolation and transcriptional characterization of mouse perivascular astrocytes
Source: PLoS One. 2020 Oct 8;15(10):e0240035. doi: 10.1371/journal.pone.0240035 (PMC7544046; doi:10.1371/journal.pone.0240035)
Supplement: S8 Table — (DOCX) [file pone.0240035.s014.docx]

**S8 Table. The 20 most enriched genes in cell cluster 7 from scRNAseq.**

| **Gene** | **p_val** | **avg_logFC** | **pct.1** | **pct.2** | **p_val_adj** | **cluster** |
| --- | --- | --- | --- | --- | --- | --- |
| *Ly6c1* | 3.11E-297 | 2.631581 | 0.757 | 0.003 | 5.75E-293 | 7 |
| *Cldn5* | 2.64E-276 | 3.0333 | 0.676 | 0.002 | 4.88E-272 | 7 |
| *Ly6a* | 1.53E-267 | 2.610614 | 0.676 | 0.003 | 2.83E-263 | 7 |
| *Flt1* | 4.15E-229 | 2.229844 | 0.595 | 0.003 | 7.66E-225 | 7 |
| *Rgs5* | 2.20E-190 | 3.249185 | 0.73 | 0.011 | 4.05E-186 | 7 |
| *Kdr* | 2.13E-175 | 1.519973 | 0.405 | 0.001 | 3.93E-171 | 7 |
| *Abcb1a* | 3.09E-173 | 1.797248 | 0.378 | 0 | 5.71E-169 | 7 |
| *Slco1a4* | 2.95E-166 | 2.095995 | 0.459 | 0.003 | 5.45E-162 | 7 |
| *Ptprb* | 5.58E-153 | 1.68062 | 0.459 | 0.004 | 1.03E-148 | 7 |
| *Higd1b* | 5.60E-147 | 1.48356 | 0.324 | 0 | 1.03E-142 | 7 |
| *Itm2a* | 1.56E-145 | 3.215614 | 0.703 | 0.016 | 2.87E-141 | 7 |
| *Cxcl12* | 1.95E-142 | 2.409864 | 0.568 | 0.009 | 3.59E-138 | 7 |
| *Foxq1* | 5.92E-133 | 0.927211 | 0.27 | 0 | 1.09E-128 | 7 |
| *Igfbp7* | 6.19E-126 | 2.372076 | 0.514 | 0.009 | 1.14E-121 | 7 |
| *Adgrf5* | 1.80E-123 | 1.023276 | 0.297 | 0.001 | 3.32E-119 | 7 |
| *Sema3c* | 1.22E-114 | 1.46279 | 0.297 | 0.001 | 2.26E-110 | 7 |
| *Emcn* | 9.06E-108 | 1.292218 | 0.243 | 0 | 1.67E-103 | 7 |
| *Slc22a8* | 5.76E-107 | 1.395455 | 0.297 | 0.002 | 1.06E-102 | 7 |
| *Car4* | 6.49E-107 | 1.457556 | 0.297 | 0.002 | 1.20E-102 | 7 |
| *Clic5* | 1.16E-106 | 0.952535 | 0.216 | 0 | 2.14E-102 | 7 |
