# Supplementary material for: Isolation and transcriptional characterization of mouse perivascular astrocytes
Source: PLoS One. 2020 Oct 8;15(10):e0240035. doi: 10.1371/journal.pone.0240035 (PMC7544046; doi:10.1371/journal.pone.0240035)
Supplement: S9 Table — (DOCX) [file pone.0240035.s015.docx]

**S9 Table. The top 10 signaling pathways in PAs as determined by Gene Set Enrichment Analyses (GSEA).**

| **Pathway** | **Normalized Enriched Value (NES)** |
| --- | --- |
| HALLMARK_EPITHELIAL_MESENCHYMAL_TRANSITION | 1.97 |
| HALLMARK_NOTCH_SIGNALING | 1.64 |
| HALLMARK_ADIPOGENESIS | 1.62 |
| HALLMARK_GLYCOLYSIS | 1.61 |
| HALLMARK_WNT_BETA_CATENIN_SIGNALING | 1.60 |
| HALLMARK_CHOLESTEROL_HOMEOSTASIS | 1.53 |
| HALLMARK_ANGIOGENESIS | 1.41 |
| HALLMARK_FATTY_ACID_METABOLISM | 1.39 |
| HALLMARK_HYPOXIA | 1.34 |
| HALLMARK_HEDGEHOG_SIGNALING | 1.05 |
