# Supplementary material for: Isolation and transcriptional characterization of mouse perivascular astrocytes
Source: PLoS One. 2020 Oct 8;15(10):e0240035. doi: 10.1371/journal.pone.0240035 (PMC7544046; doi:10.1371/journal.pone.0240035)
Supplement: S10 Table — (DOCX) [file pone.0240035.s016.docx]

**S10 Table. The top 30 genes with enriched expression in PAs versus non-PAs based on bulk RNAseq.**

| **Gene Symbol** | **Description** | **P-value** |
| --- | --- | --- |
| *Alpk1* | Alpha-protein kinase 1 enzyme | 1.73E-13 |
| *Fam228a* | Family with sequence similarity 228a | 1.11E-10 |
| *Zfp783* | Zinc finger protein 783 | 4.17E-10 |
| *Sema4a* | Semaphorin-4A | 6.81E-10 |
| *Serpinh1* | Serpin family h member 1 | 5.43E-09 |
| *Fzd2* | Frizzled class receptor 2 | 5.43E-09 |
| *Lcat* | Lecithin cholesterol acyltransferase | 1.07E-08 |
| *Oaf* | Out at first | 2.25E-08 |
| *Lfng* | Lunatic fringe | 2.82E-08 |
| *Itga7* | Integrin subunit alpha-7 | 3.31E-08 |
| *Entpd2* | Ectonucleoside triphosphate diphosphohydrolase 2 | 5.11E-08 |
| *P4ha2* | Prolyl 4-hydroxylase subunit alpha-2 | 6.39E-08 |
| *Sema4b* | Semaphorin-4B | 6.95E-08 |
| *Ppp1r3d* | Protein phosphatase 1 regulatory subunit 3d | 8.45E-08 |
| *Fam20a* | Family with sequence similarity 20 | 1.59E-07 |
| *Reg2* | Regenerating islet-derived 2 | 1.67E-07 |
| *Als2cl* | Als2 c-terminal like protein | 2.10E-07 |
| *Alpl* | Alkaline phosphatase | 3.49E-07 |
| *Prelp* | Proline/arginine-rich end leucine-rich repeat protein | 3.49E-07 |
| *Agt* | Angiotensin | 3.62E-07 |
| *Micall2* | Mical like protein 2 | 5.21E-07 |
| *Gjb2* | Gap junction beta-2 protein | 9.19E-07 |
| *Per3* | Period circadian regulator 3 | 1.01E-06 |
| *Lama5* | Laminin subunit alpha 5 | 1.53E-06 |
| *Gdf10* | Growth differentiation factor 10 | 3.67E-06 |
| *Fjx1* | Four-jointed box kinase 1 | 6.52E-05 |
| *Lama3* | Laminin subunit alpha 3 | 1.45E-04 |
| *Frem2* | Fras related extracellular matrix 1 | 1.48E-04 |
| *Frem1* | Fras related extracellular matrix 1 | 3.43E-04 |
| *Fstl1* | Follistatin like 1 | 4.24E-04 |
